# Diversity of plant assemblages dampens the variability of the growing season phenology in wetland landscapes

**DOI:** 10.1186/s12862-021-01817-6

**Published:** 2021-05-19

**Authors:** Guillaume Rheault, Esther Lévesque, Raphaël Proulx

**Affiliations:** 1 Centre de Recherche sur les Interactions Bassins Versants-Écosystèmes Aquatiques (RIVE), Département des sciences de l’environnement, Université du Québec à Trois-Rivières, 3351 Boulevard des Forges, Trois-Rivières (Québec), G8Z 4M3 Canada; 2 Chaire de Recherche en Intégrité Écologique, Département des Sciences de l’environnement, Université du Québec à Trois-Rivières, 3351 Boulevard des Forges, Trois-Rivières (Québec), G8Z 4M3 Canada; 3 Centre d’Études Nordiques, Pavillon Abitibi-Price, Université Laval, 2405 rue de la Terrasse, Québec (Québec), G1V 0A6 Canada

**Keywords:** Plant phenology, Diversity stability relationship, Biodiversity ecosystem functioning, Temporal asynchrony, Marshes, Bogs, Wet meadows

## Abstract

**Background:**

The functioning of ecosystems is highly variable through space and time. Climatic and edaphic factors are forcing ecological communities to converge, whereas the diversity of plant assemblages dampens these effects by allowing communities’ dynamics to diverge. This study evaluated whether the growing season phenology of wetland plant communities within landscapes is determined by the climatic/edaphic factors of contrasted regions, by the species richness of plant communities, or by the diversity of plant assemblages. From 2013 to 2016, we monitored the phenology and floristic composition of 118 wetland plant communities across five landscapes distributed along a gradient of edaphic and climatic conditions in the Province of Québec, Canada.

**Results:**

The growing season phenology of wetlands was driven by differences among plant assemblage within landscapes, and not by the species richness of each individual community (< 1% of the explained variation). Variation in the growing season length of wetlands reflected the destabilizing effect of climatic and edaphic factors on green-up dates, which is opposed to the dampening effect of plant assemblage diversity on green-down dates.

**Conclusions:**

The latter dampening effect may be particularly important in the context of increasing anthropogenic activities, which are predicted to impair the ability of wetlands to adapt to fluctuating environmental conditions. Our findings suggest that stakeholders should not necessarily consider local species-poor plant communities of lower conservation value to the global functioning of wetland ecosystems.

**Supplementary Information:**

The online version contains supplementary material available at 10.1186/s12862-021-01817-6.

## Background

The spatial and temporal variability of ecosystems has received a great deal of attention in the last decades in the context of biodiversity loss, climate change and their impact on ecosystem functioning. Ecosystem functioning is known to vary (i) among landscapes due to their environmental context, especially edaphic and climatic conditions [1]; (ii) within landscapes because individual communities respond differently to local conditions at the landscape level [2–5]; (iii) inter-annually due to temporal variation in edaphic and climatic conditions [1, 5]; and (iv) within communities because individual species respond differently to local conditions at the community level [6–10]. In temperate and arctic regions, factors such as climate constrain the onset and offset of ecosystem processes [1]. During the growing season, competition for resources induces species-specific responses to environmental conditions, which may drive the observed variation in growth among ecological communities within landscapes and dampen the effect of edaphic and climatic conditions [4, 11]. The relative importance of these different sources of variation on ecosystem functioning has yet to be quantified in natural conditions and the dampening effect of the diversity of plant assemblages remains to be investigated.

Plant phenology is a key functional trait that links growth and reproduction events to the functioning of ecosystems [1]. Over large geographic extents, phenology is driven by the effect of climatic and edaphic factors on plant growth and stress tolerance [12–14]. In turn, plant phenology determines several ecosystem functions, such as pollination [15], herbivory [16] and carbon uptake [17]. One main advantage of studying plant phenology is that the timing of biological events can be monitored at high spatial and temporal resolution through satellite or time-lapse imagery [18, 19].

Although climatic and edaphic factors are important determinants of plant phenology at both large and small observational scales, recent studies also emphasized the importance of plant species richness and composition on phenology [20–22]. Species subjected to similar climatic and edaphic conditions tend to show large inter-specific differences in their phenology [19, 23–26]. For example, Wilsey et al. [26] compared grassland communities in northern latitudes and found that their growing season length differed by nearly 40 days. A study by Meng et al. [24] reported large inter-annual variations in the flowering sequence (i.e., ranking order) of 15 co-occurring plant species. Thus, the biodiversity of plant assemblages could be an important driver of plant phenology by introducing spatial variability and temporal asynchrony between communities within landscapes.

Conservation biologists use estimates of plant species richness to characterize temporal changes in the ecological dynamics of both ecological communities and landscapes. Yet, the role of plant species richness on the regulation of plant phenology was investigated in a few cases only. A lengthening of the growing season with increasing plant species richness at the landscape level was observed across six biogeographic regions of central Europe, independently of altitude and land-use descriptors [21]. Rheault et al. [22] monitored 28 wetland plant communities and showed that the growing season length was, on average, 30 days longer in species-rich communities. However, the latter authors noted that the relationship between plant species richness and growing season length was contingent on the climatic conditions [22]. Studies of plant phenology have yet to disentangle the relative importance of species richness, community asynchrony, and community temporal variance on the dynamics of ecosystems.

The coefficient of variation of an ecosystem function (e.g., aerial biomass, growing season length) measured on several occasions is a standard measure of temporal variation; i.e., the reciprocal of stability. Using this metric, three key variables determine the temporal variability of a plant community [27]: (i) species asynchrony, (ii) species temporal variance and (iii) species average functioning. Species asynchrony is a measure of how temporally de-correlated is the functioning of each species relative to the others in the community. The variability of a plant community will be low (i.e., stability will be high) if species asynchrony is high and if species temporal variance is low [27]. The above principles can be scaled-up to the landscape level such that the variability is this time determined by: (i) community asynchrony, (ii) community temporal variance and (iii) community average functioning. For the diversity of plant assemblages to stabilize the functioning of ecosystems, the expectation is that community asynchrony is an important determinant, while community temporal variance is comparatively less important. High spatial variability in the average functioning of communities is also stabilizing because it buffers differences among landscapes.

**Fig. 1:**
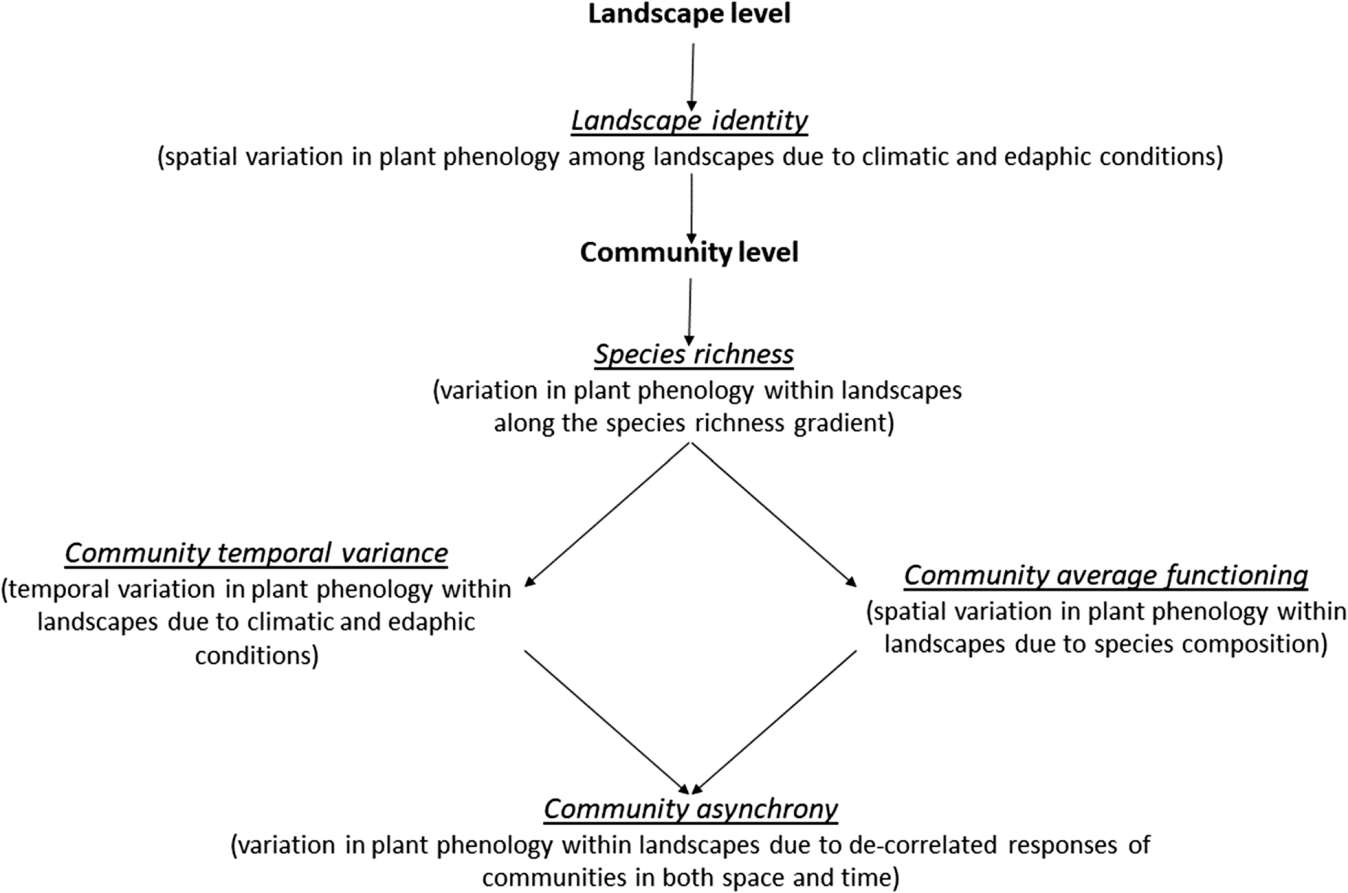
Nested design of this study and definition of each component. *Species richness*, *Community average functioning*, *Community temporal variance* and *Community asynchrony* are all nested within *Landscape identity*

**Fig. 2:**
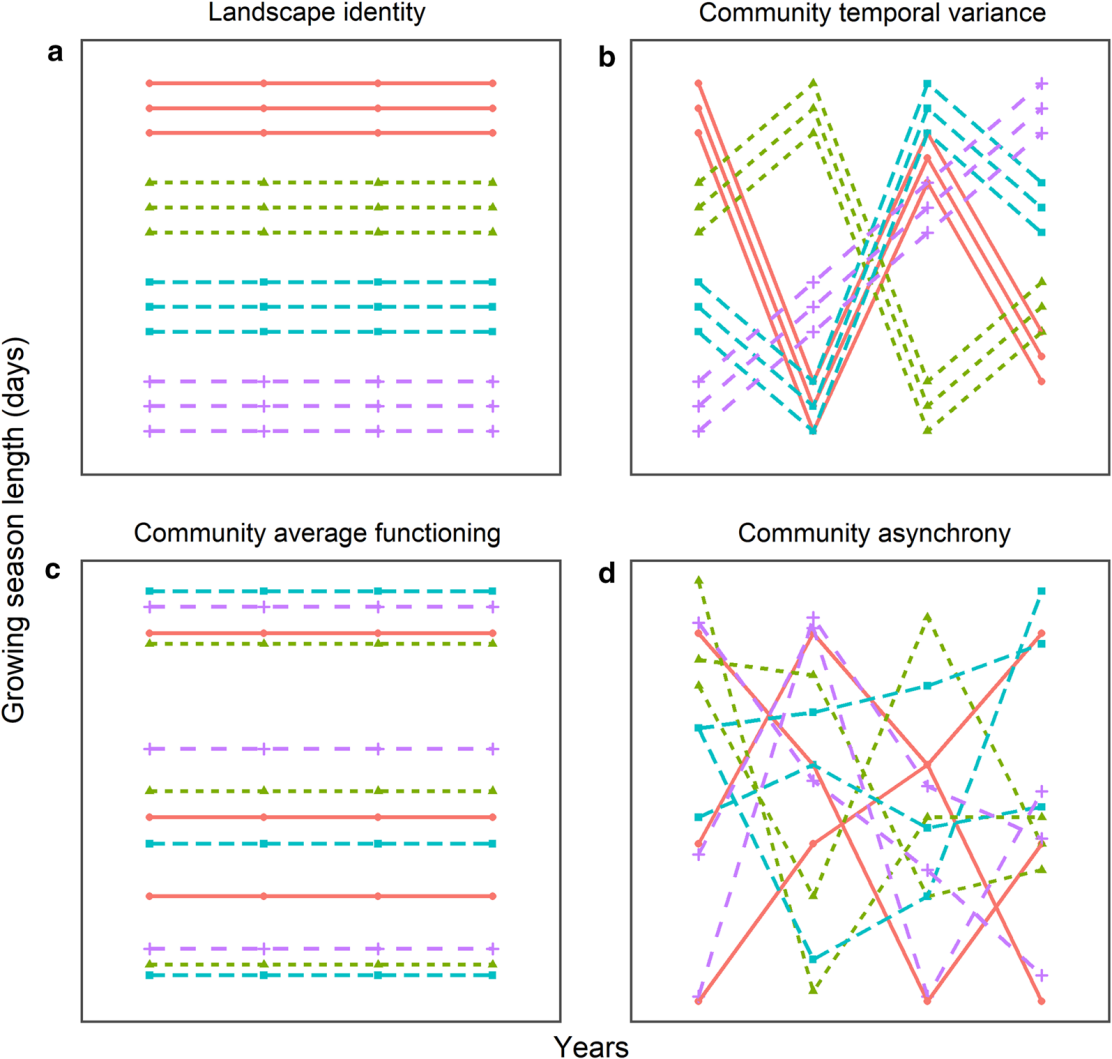
Contribution of different components to the growing season length of simulated plant communities: **a** *Landscape identity*, **b** *Community temporal variance*, **c** *Community average functioning* and **d** *Community asynchrony*. In each scenario, lines represent plant communities and colors represent different landscapes. Each dot represents the growing season length of a unique community for a given year, whereas each line shows its inter-annual trend. Scenarios (**a**) and (**b**) are destabilizing because communities respond to the climatic and edaphic factors that characterize each landscape each year, which increase variation in the growing season length among landscapes. In contrast, scenarios (**c**) and (**d**) are stabilizing because the diversity of plant assemblages averages the variation among landscapes. The percent of variation explained by one component is near 100% in each of the above scenarios

The objective of this study was to evaluate whether the growing season phenology of wetland plant communities within landscapes is determined by climatic and edaphic factors, by the diversity of species within plant communities, or by the diversity of plant assemblages among communities. The approach that we developed in this paper consists of partitioning the growing season phenology of plant communities (green-up and green-down dates, and growing season length) into five components using linear models: (i) *Landscape identity*, (ii) *Community temporal variance*, (iii) *Community average functioning*, (iv) *Species richness* and (v) *Community asynchrony*. We present a schematic view of the nested design of our study (Fig. 1) and the partitioning of the variation in plant phenology components (Fig. 2). Disentangling between these alternative scenarios is critical because they involve different management scales and policies. We also provide a direct test of the diversity-stability relationship using the species richness of individual communities as a measure of plant diversity and the coefficient of variation of plant phenology as a standard measure of temporal variation.

## Results

We successfully established the long-term SAuVER network, for Surveillance Automatisée de la Végétation des Écosystèmes Riverains, which monitors the seasonal phenology of 118 plant communities across five wetland landscapes that differ in their climatic and edaphic characteristics. Mean annual temperature in each of these landscapes ranged from − 4.6 to 6.4 °C, mean total annual precipitation from 661 to 1085 mm, soil pH from 3.5 to 6.4 and soil moisture from 40% to nearly 80% (Table 1). Mean green-up dates varied by almost a month among landscapes, but mean green-down dates varied by no more than 12 days. The growing season length of plant communities was on average 33 days longer at lower latitudes (Table 1).

**Table 1:** Environmental context of the five wetland landscapes

	Scirbi	Maskinongé	Lac-à-la-tortue	Bog-à-lanières	Umiujaq
Type	Wet meadows	Fluvial marshes	Peatlands	Peatlands	Wet meadows
Latitude	46.07	46.19	46.55	47.59	56.57
Nbr. Comm.	20	30	20	20	28
Temperature	6.40	5.10	4.80	2.00	− 4.60
Precipitation	997.2	1009.3	1085.0	1016.4	660.8
Soil pH	5.48 ± 0.26	6.36 ± 0.28	3.47 ± 0.11	4.01 ± 0.11	5.38 ± 0.38
Soil moisture	39 ± 14	55 ± 15	78 ± 11	60 ± 13	58 ± 20
Green-up	160 ± 15	164 ± 8	147 ± 9	157 ± 7	172 ± 7
Green-down	266 ± 11	270 ± 15	263 ± 11	256 ± 8	258 ± 6
GSL	106 ± 15	112 ± 15	117 ± 12	99 ± 11	84 ± 10

Wetland type (Type), latitudinal location (Latitude; Decimal Degrees), number of surveyed plant communities (Nbr. Comm.), mean annual temperature (Temperature; °C), mean total annual precipitation (Precipitation; mm), soil pH (Soil pH; Mean ± Sd), soil moisture (Soil moisture; %, Mean ± Sd), green-up date (Green-up, day of year, Mean ± Sd), green-down date (Green-down, day of year, Mean ± Sd), growing season length (GSL; number of days elapsed between Green-up and Green-down dates, Mean ± Sd). Mean and Sd values for soil pH and soil moisture were calculated from four measures taken once (August 2015) in each plant community. Mean and Sd values for green-up, green-down dates and growing season length were calculated across all communities within each landscape over the period 2013–2016

Using a hierarchical partitioning approach, we determined that *Landscape identity* was the most influential component for green-up dates, emphasizing the influence of climatic and edaphic factors on leaf-out events (Table 2). *Community temporal variance* (year identity within landscapes) was the second most influential component on green-up dates. However, its influence diminished during the season, explaining no more than 15% of the variation in green-down dates. *Community average functioning* and *Community asynchrony* within landscapes explained the largest share of variation in green-down dates. *Species richness* explained no variation in the phenology of plant communities, whereas *Community asynchrony* explained more than 20% of green-up and green-down dates, highlighting the important role of compensatory dynamics in stabilizing the functioning of ecosystems (Table 2).

**Table 2:** Hierarchical partitioning of the variation in plant phenology

Phenophases	Landscape identity	Community temporal variance	Species richness	Community average functioning	Community asynchrony
G-U	0.42	0.27	< 0.01	0.09	0.22
G-D	0.16	0.15	< 0.01	0.34	0.35
GSL	0.46	0.08	< 0.01	0.29	0.17

Plant phenology was explained by: *Landscape identity*, *Species richness*, *Community temporal variance* (year within landscape), *Community average functioning* (community identity within landscape), and *Community asynchrony* (interaction year x community identity). The values show the coefficient of determination (R^2^) associated with each phenology component when modelling the green-up date (G-U), the green-down date (G-D) and the growing season length (GSL)

**Fig. 3:**
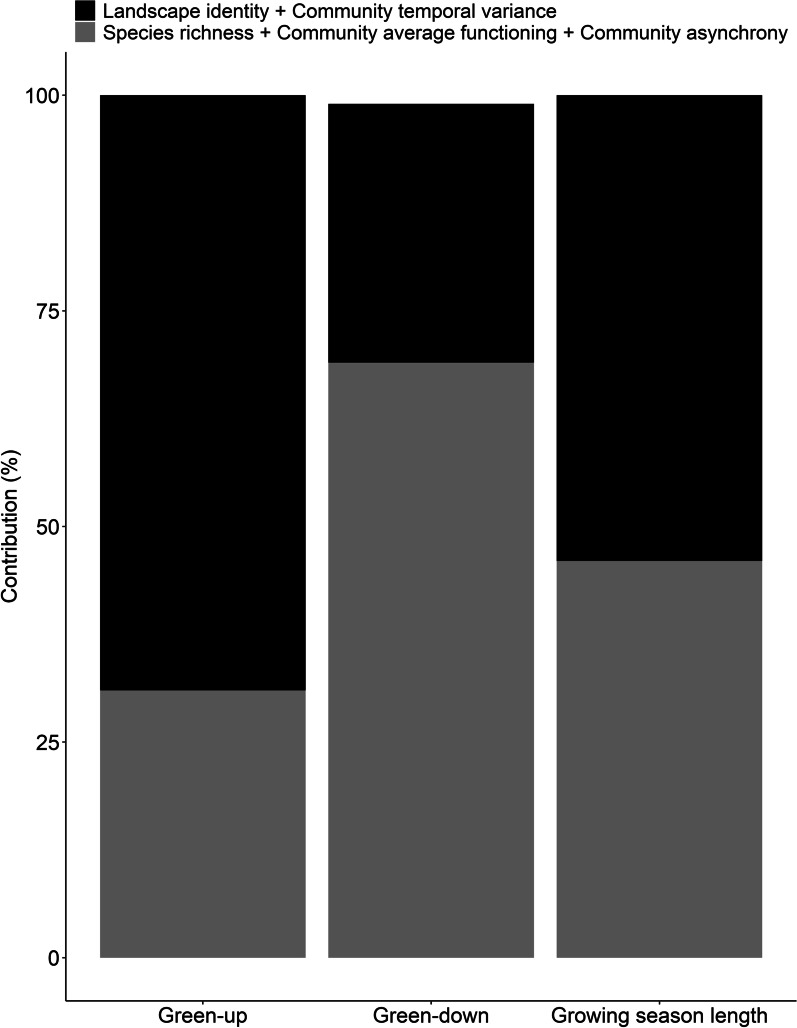
Percent relative contribution of the combined effect of *Landscape identity* and *Community temporal variance* (grey) and the combined effect of *Species richness*, *Community average functioning* and *Community asynchrony* (black) to the green-up and green-down dates, as well as the growing season length of 118 plant communities. The relative contribution of *Landscape identity* and *Community temporal variance* are summed to represent the effect of climatic and edaphic factors on plant phenology, while *Species richness*, *Community average functioning* and *Community asynchrony* are summed to represent the effect of plant assemblage diversity on phenology

Climatic and edaphic factors, represented by the combination of *Landscape identity* and *Community temporal variance*, contributed to more than two thirds (69%) of the variation in green-up dates. The result was reversed when considering the diversity of plant assemblages within landscapes, represented by *Community average functioning* and *Community asynchrony*, which together explained 69% of the variation in green-down dates. The growing season length of wetland landscapes reflected the destabilizing effect of climatic/edaphic factors on green-up dates and the stabilizing effect of the diversity of plant assemblages on green-down dates (Fig. 3).

**Fig. 4:**
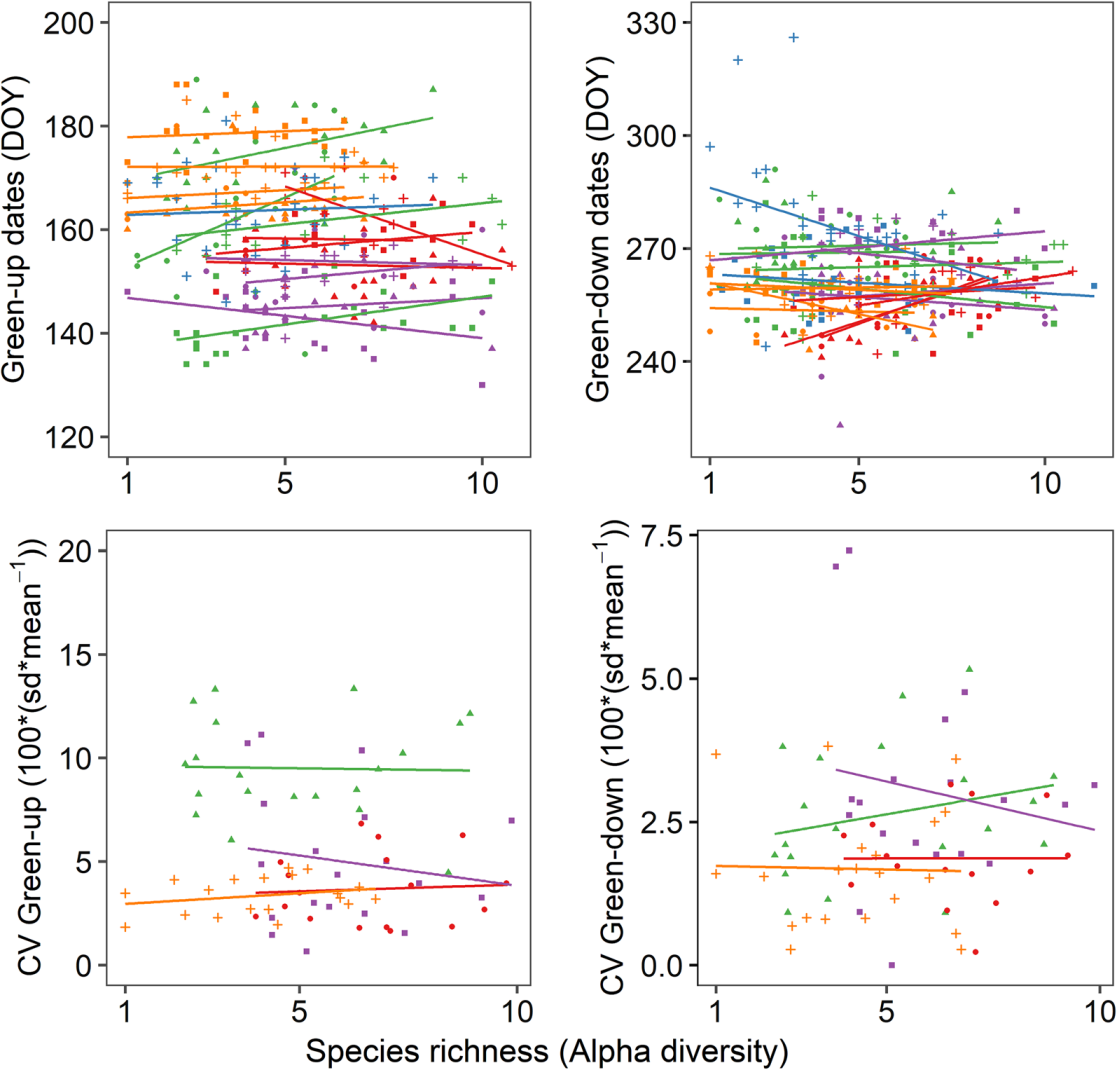
Species richness relationships to the Green-up (left) and Green-down (right) phenology of plant communities for every combination of year and landscape. Each dot represents the observed day-of-year (DOY) phenology of a unique plant community (top panels), or its temporal fluctuation (coefficient of variation; CV) across years (bottom panels). Each color represents a different landscape: Scirbi (Green), Maskinongé (Blue), Lac-à-la-Tortue (Purple), Bog-à-lanières (Red), Umiujaq (Orange). In the upper panels, lines of the same color represent different years. In the lower panels, none of the relationships between plant species richness and inter-annual CV is statistically noteworthy (p > 0.05, R^2^ < 0.01)

Hierarchical partitioning of the variation unveiled the weak contribution of plant species richness in explaining the growing season phenology of plant communities. Among all possible combinations of years and landscapes, we did not find consistent relationships between plant species richness and growing season phenology (Table 2). Moreover, we did not find any evidence of a relationship between the average *Species richness* of a plant community and the inter-annual temporal instability of green-up or green-down dates (Fig. 4, lower panels). To summarize, neither the phenology nor the inter-annual stability of a plant community was determined by its species richness (Fig. 4).

## Discussion

The growing season length of wetlands opposed the destabilizing effect of climatic and edaphic factors on green-up dates to the stabilizing effect of the diversity of plant assemblages on green-down dates. Climatic and edaphic factors, expressed through *Landscape identity* and *Community temporal variance*, explained a larger relative proportion of the variation in green-up dates across landscapes and years. Conversely, the diversity of plant assemblages, expressed through *Community average functioning* and *Community asynchrony*, explained a larger relative proportion of the variation in green-down dates, thus revealing a seasonal shift in the factors that drive the phenology of wetland landscapes. Climatic and edaphic factors are destabilizing because they force plant communities in a given landscape, in a given year, to converge towards a similar phenology. In this context, the diversity of plant assemblages is stabilizing because it allows plant communities to diverge into a portfolio of growth phenology patterns over space and time.

In theory, species richness could increase the average functioning and temporal stability of a plant community by increasing species asynchrony and decreasing temporal variance [27]. Here, we found no influence of species richness on the growing season phenology of 118 plant communities. Nevertheless, our results show that growing season length varied by nearly one month between plant communities subjected to similar climatic and edaphic conditions (i.e., within the same landscape). We propose that the diversity of plant assemblages supports the growing season phenology and temporal stability of wetland landscapes, irrespective of the species richness of local communities. A recent study of 78 plant communities in two wetland landscapes revealed a consistently strong negative relationship between the uniqueness and species richness of plant communities [28]. Hence, unique assemblages of plant species in wetland landscapes are often species poor. These unique assemblages not only contribute to the diversity of plant assemblages, but also may be key to the resilience of wetlands.

*Landscape identity* and *Community temporal variance* explained most of the variation in green-up dates among plant communities of the SAuVER network. Because this represents the fraction of variation attributed to climatic and edaphic factors, and not the diversity of plant assemblages, it is also harder to manage locally. The destabilizing effect of climatic and edaphic factors on green-up dates may be related to the large latitudinal gradient covered by the SAuVER monitoring network. In high-latitude landscapes, climatic factors control the activation of plant metabolism and growth onset [12, 29, 30]. In the specific case of wetlands, snowmelt and flooding events determine the light available at the ground level and temperature profiles, as well as O_2_ availability to plants [31, 32]. Given that spring temperatures, snowmelt dates, flooding amplitude and duration not only vary over space, but also from year to year, factors such as species composition and richness only have a weak influence on green-up dates in comparison to climatic and edaphic factors.

Our findings showed that maintaining a diversity of plant assemblages dampens spatial and temporal variations in the growing season length of wetland landscapes. Previous studies have underlined the broad range of variation in the green-down dates of plant communities within landscapes [20, 22]. Such variations in plant phenology reflect community-specific responses and adaptations to a similar set of climatic and edaphic conditions. Said otherwise, the broad range of green-down dates observed within a given region is largely driven by the ecology of plant communities. Our analyses, however, did not disentangle community-specific from species-specific responses to climatic and edaphic factors. A critical question in this context is whether a few keystones, but functionally redundant, plant species in the landscape drive the green-down phenology of plant communities [33]. A better understanding of the phenological strategy of each individual species will be required to tackle this question in greater depth.

Community asynchrony consistently explained 20–40% of the variation in the growing season length of plant communities, revealing the key role of compensatory dynamics in stabilizing wetlands’ functioning. The early green-down of some plant assemblages in a given year was compensated by the late green-down of other plant assemblages, and vice versa in other years. While previous studies identified species asynchrony as a key principle for dampening the functioning of local patches of vegetation over time (e.g., [8, 10]), our results suggest that community asynchrony may be just as important for dampening the functioning of whole landscapes. Our findings did not support the hypothesis that local species richness stabilizes the functioning of plant communities, which contrasts with the results commonly reported from other biodiversity experiments (e.g.: [34, 35]). Among all possible combinations of years and landscapes, we did not find a consistent relationship between plant species richness and plant phenology. Neither did we find evidences of a stabilizing effect of plant species richness on inter-annual fluctuations (i.e., temporal CV) in the growing season length of plant communities.

There is a growing body of literature emphasizing the importance of conserving a diversity of plant assemblages and not only high levels of local plant species richness. Dampening of ecosystem functioning through community asynchrony was so far only hypothesized by theoretical models and revealed in experimental grasslands [4, 5, 36, 37]. The present study reports and replicates this dampening principle on a large network of freely assembled plant communities spanning several wetland types. We showed that the community asynchrony principle operates independently of the climate and edaphic factors that prolong, or constrain, the growing season of plant communities. For instance, climate warming in temperate and boreal landscapes should be associated to a longer growing season (e.g.: [38, 39]). Maintaining a diversity of plant assemblages may offer this insurance mechanism (*aka* portfolio effect; [3]) to the functioning of ecosystems in the face of rapid environmental changes.

## Conclusions

We revealed that dampening of temporal variation in the growing season of wetlands comes from the diversity of plant assemblages and their asynchronous responses, and not from maximizing the species richness of each individual community. While comparable in duration to other diversity-stability experiments (e.g., [40–43]), we acknowledge that the observed variation in plant phenology is limited to only four years of data. Yet, the strength of the SAuVER network stems from monitoring contrasted landscapes using standard protocols. Years 2013–2016 were also not exceptional in terms of climatic conditions in the Quebec Province, so we are confident that our results will generalize on the long term. Dampening the functioning of ecosystems by maintaining a diversity of plant assemblages may be key in the context of increasing anthropogenic activities, which may impair the ability of wetlands to adapt to fluctuating environmental conditions [44, 45]. Stakeholders should not necessarily consider species-poor plant communities as of lower conservation value to the global functioning of ecosystems. This is particularly true of wetland landscapes, where local patches of vegetation are often naturally dominated by a few species. A reconsideration of conservation strategies is warranted to prioritize the conservation of natural wetland dynamics and the environmental heterogeneity that together promote the diversification of plant assemblages within landscapes.

## Methods

### Experimental design

Wetlands represent an ideal system for studying the dampening effect of plant diversity on ecosystem functioning, as these landscapes show large inter-annual variations in their growing season phenology and a high species turnover in space and time [22]. In 2013, we established a long-term monitoring network, called SAuVER, to monitor the taxonomic assemblage and growing season length of 118 plant communities across five wetland landscapes; e.g.: arctic wet meadows, two peatland ecosystems, fluvial marshes and temperate wet meadows. Plant communities were monitored from 2013 to 2016 in the five landscapes spatially distributed between 46° N and 56° N in Quebec, Canada (See Additional file 1: Figure S1). To minimize the effect of environmental heterogeneity on the phenology of plant communities in each landscape, we selected, within an area of less than one square kilometer, 20–30 plant communities composed of herbaceous and low-shrub vegetation. We locally paired plant communities dominated by one or two species with nearby species-rich communities to create a species richness gradient that was independent of local environmental conditions (See Additional file 1: Figure S2 and Table S1). Differences in phenology among plant communities within landscapes reflected differences in their species composition. Plant diversity components were defined by both the number of species present (i.e.; species richness) and its unique species composition (i.e., community identity). Our experiment allowed us to evaluate the independent contribution of local species richness and community identity on plant phenology. By design, the SAuVER network emphasizes a gradient in species richness and diversity of plant assemblages within each landscape.

We used Wingscape timelapse cameras (Wingscape®, Albaster, USA) to monitor changes to the species assemblages (richness and identity), and the growing season phenology of plant communities. We programmed each camera to take three pictures per day (9 a.m., 12 p.m. and 3p.m.), from April to December at lower latitudes, and from June to October at higher latitudes. The size of each image was 2592 × 1944 pixels and images were stored in JPEG format (RGB images). We left the cameras in the field the whole season, except in the fluvial marsh landscape where we took pictures on a weekly basis to prevent poaching. In the latter, we mounted the camera on a metal post and followed the same procedure used in the other landscapes. We positioned the cameras at a height of 1.3 m in peatlands and Arctic wet meadows, and 1.5 m in fluvial marshes and temperate wet meadows. Each camera was pointing downward towards the vegetation with an angle of 45 degrees, capturing a ground area of approximately 16 m^2^ [22].

### Field measurements

We conducted image-based taxonomic surveys of the overstory vegetation in each community through a visual assessment of four pictures taken on the 15th day of each month in June, July, August and September. We built a presence-absence community matrix and counted the total number of species present in the overstory of each plant community each year. To guide the identification process, we referred to an exhaustive botanical survey conducted in each ecosystem on a yearly basis. A pilot study of temperate wet meadows showed that the image-based taxonomic identification of plant species richness was highly correlated to field surveys [22]. Species richness of each community, each year, was determined as the mean number of observed species in the four samples. We used this variable in the variance partitioning procedure described below.

### Plant phenology

We assessed the growing season length of plant communities using an automatic R procedure [46, 47]. In each image, we calculated a green chromatic coordinate index (G_CC_) using the following equation:

1 $$Gcc = \frac{{\text{G}}}{{{\text{R}} + {\text{G}} + {\text{B}}}}$$

where R, G and B represented average red, green and blue pixels’ digital numbers of each image [48]. We created Gcc time series for each community and year by assigning the median (50th percentile) of all available Gcc values within a non-overlapping moving window using the *medianFilter* function from “FBN” package [49, 50]. To extract the Gcc seasonal trend of each community, we applied a cubic smoothing spline function on each filtered time series using the *gam* function of the “mgcv” package [50]. The smoothing parameter used for each time series was determined automatically by minimizing the generalized cross-validation score. We then implemented the method proposed by White et al. [51] in which green-up and green-down dates are determined by applying a threshold to the smoothing function. We used 50% of the rescaled greenness range as a threshold value. Green-up and green-down dates were found when Gcc increased above or decreased below the threshold value, respectively ([51], See Additional file 1: Figure S3). We defined the growing season length of each plant community as the number of days elapsed between the green-up and green-down dates. After removing time series that could not be used due to malfunctioning cameras, or modification to the field of view by wildlife (mostly moose and black bears), we ended up with 306 and 324 time series for green-up and green-down dates, respectively, and 286 complete time series that could be used to assess the growing season length (See Additional file 1: Tables S2 and S3).

### Statistical analyses

We performed a hierarchical partitioning of the variation to assess the contribution of *Landscape identity* and *Community temporal variance*, as well as *Species richness, Community average functioning,* and *Community asynchrony* on the growing season phenology of plant communities. Specifically, we calculated the proportion of the total variance explained by each independent variable for each of the three phenology variables (green-up and green-down dates, and growing season length) using linear models and the *lm* function in R [46]. We introduced each independent variable successively to account for the hierarchical structure of our data: *Landscape identity* and *Species richness,* followed by *Community temporal variance*, *Community average functioning* and *Community asynchrony,* with the last three variables nested within *Landscape identity* (see Additional file 1, for an example). *Landscape identity* and *Species richness* are part of the SAuVER experimental design and were fitted first. We determined the contribution of *Species richness* after removing the contribution of *Landscape identity*. We modelled *Community temporal variance* and *Community average functioning* (both nested within *Landscape identity*) using year (2013, 2014, 2015, 2016) and community identity (1:118), respectively (Fig. 2). Finally, we modelled *Community asynchrony* as the interaction between year and community identity, thus capturing the residual variation associated with the de-correlated temporal dynamics of plant assemblages within landscapes (Fig. 2). We included all components as factors (unordered levels), with the exception of *Species Richness* (continuous scale). Partitioning of the variation in plant phenology was purely additive such that the variance explained by the different components always sums to one. Including variables sequentially in the models may slightly underestimate the contribution of *Community average functioning* and *Community asynchrony*. However, fitting all terms simultaneously did not change the results, which indicated that there is no shared variation between variables.

At last, we directly tested whether plant *Species richness* increased the temporal stability (i.e., decreased year-to-year fluctuations) of plant phenology at the community level. This represents a standard test of the diversity-stability relationship at the level of plant communities. We used the coefficient of variation (CV) as a measure of instability [52, 53]:

2 $${\text{CV}} = 100 \times {{{\text{SD}}} \mathord{\left/ {\vphantom {{{\text{SD}}} {{\text{Mean}}}}} \right. \kern-\nulldelimiterspace} {{\text{Mean}}}}$$

where SD represents the standard deviation of a phenology variable within one community and Mean represents the arithmetic average across years over the 2013–2016 period. We used the average number of species observed within each unique plant community for the period 2013–2016 as our measure of *Species richness* for this analysis. We tested for a linear relationship between the plant species richness of a community and the CV of green-up and green-down dates. We excluded the fluvial marsh landscape from this analysis because we did not have four years of data for all communities. Linear models and hierarchical partitioning procedures were performed in R 3.4.0 [46].

## Supplementary Information

**Additional file 1:** Provides details on the study design (Figure S1 and Figure S2), spatial and temporal variations in the growing season length (Figure S3, Figure S4 and Figure S5) and influence of edaphic conditions on plant phenology (Table S1 and Table S2). The raw data used in the present study are available in Table S3.

## Data Availability

All data generated or analysed during this study are included in this published article and its supplementary information files.

## Abbreviations

CV: Coefficient of variation
G_cc_: Green chromatic coordinate index
G-D: Green-Down
GSL: Growing season length
G-U: Green-up
Sd: Standard deviation
