# Supplementary material for: Diversity of plant assemblages dampens the variability of the growing season phenology in wetland landscapes
Source: BMC Ecol Evol. 2021 May 19;21:91. doi: 10.1186/s12862-021-01817-6 (PMC8136205; doi:10.1186/s12862-021-01817-6)
Supplement: Supplementary file 1 — Additional file 1: Provides details on the study design (Figure S1 and Figure S2), spatial and temporal variations in the growing season length (Figure S3, Figure S4 and Figure S5) and influence of edaphic conditions on plant phenology (Table S1 and Table S2). The raw data used in the present study are available in Table S3. [file 12862_2021_1817_MOESM1_ESM.docx]

Additional informations


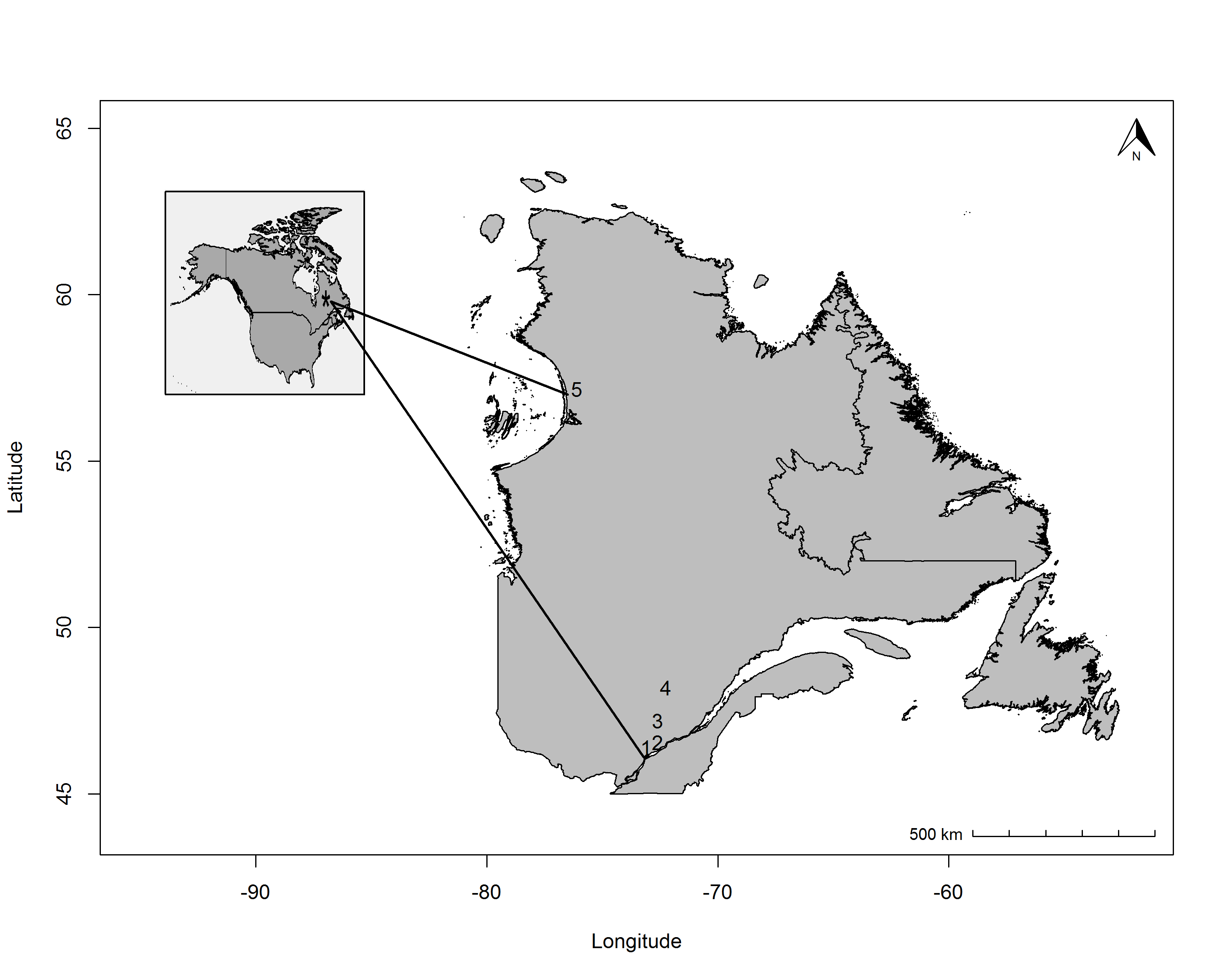


Figure S1. Latitudinal distribution of the five monitored landscapes within the SAuVER network. 1- SCIRBI: Temperate wet meadows, 2- Maskinongé: Fluvial marshes 3- Ecological reserve of Lac-à-la-Tortue; Peatlands, 4- Ecological reserve of Bog-à-lanières; Peatlands, 5- Umiujaq; Arctic wet meadows.

Environmental context of each monitored ecosystem

Climatic data were extracted from the databases maintained by the Ministère du Développement Durable et de la Lutte aux Changements Climatiques during the period 1981 to 2010 (http://www.mddelcc.gouv.qc.ca/climat/normales/). Edaphic conditions data were assessed during sampling period of August 2015. Mean pH and Moisture corresponds to the mean values of all measurement performed in each landscape which consist of four measures per plant communities in each landscape. In wet meadows and marshes, soil pH was measured in the lab from soil sample collected in the field using a 1:8 (v/v) soil:water solution. Samples were analyzed no later than one week after sampling. In ombrotrophic bogs, water pH was measured directly in the field. All pH measurements were taken using pH probe HI98121 (Hanna instrument, Smithfield, RI, USA). Soil moisture was assessed using WetSensor device (Delta-T Devices Ltd, Cambridge, UK) directly in the field. Within each landscape, field measurements were done within the same day.

Edaphic conditions relationship with species richness

In 2015, plant communities within the same landscape were similar in terms of soil and water pH, but a gradient of soil moisture was observed. However, our analysis revealed that the species richness gradient among communities selected in each landscape was independent of these edaphic conditions. Indeed, no clear relationship was observed between edaphic conditions and local species richness in each landscape (Figure S2).


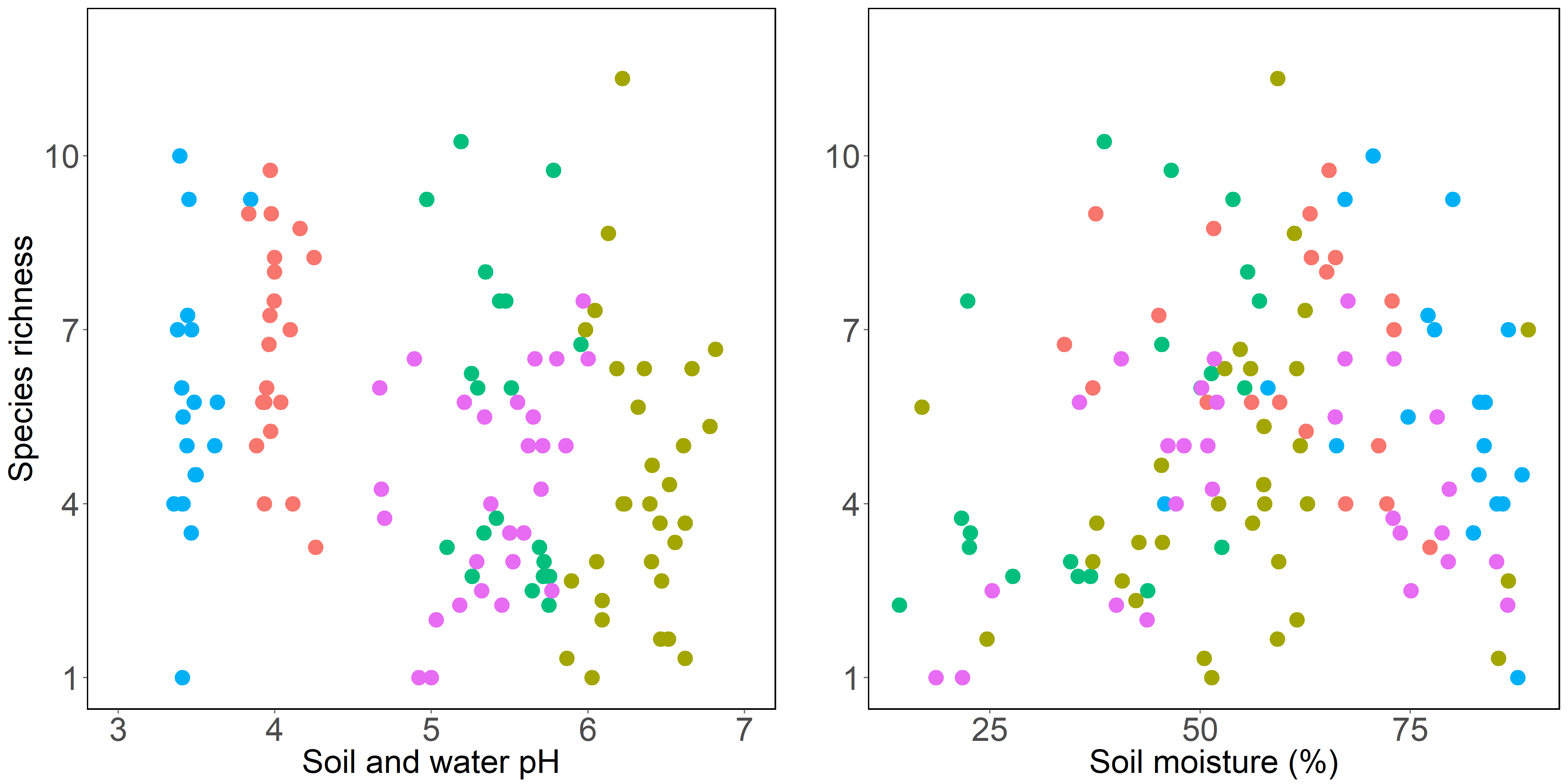


Figure S2. Edaphic conditions relationship with species richness within each landscape. pH (Left panel) and Soil moisture (Right panel) relationships with species richness (alpha diversity). 1- SCIRBI (Green), 2- Maskinongé (Brown), 3- Lac-à-la-Tortue(Red), 4- Bog-à-lanières (Blue), 5- Umiujaq (Purple).

Influence of edaphic conditions on the growing season phenology

Edaphic conditions observed in 2015 did not explain more than 10% of growing season variability, thus they did not play an important role on the regulation of plant phenology in 2015 (Table S1).

Table S1. Contribution of soil pH, soil moisture and their interactions (fixed effect; Marginal R^2^) and the landscape identity (random effect on the intercept of each phenophases; Conditional R^2^) to green-up date (Green-up), green-down date (Green-down) and growing season length (Length) in 2015.

| Phenophases | Marginal R^2^ | Conditional R^2^ |
| --- | --- | --- |
| Green-up | 0.03 | 0.93 |
| Green-down | 0.10 | 0.52 |
| Length | 0.08 | 0.85 |

Table S2. Number of time series analysed for green-up and green down dates and growing season length each year.

| **Years** | **Green-up** | **Green-down** | **Growing season length** |
| --- | --- | --- | --- |
| 2013 | 54 | 53 | 50 |
| 2014 | 81 | 67 | 63 |
| 2015 | 76 | 107 | 78 |
| 2016 | 103 | 97 | 95 |
| All years | 306 | 324 | 286 |


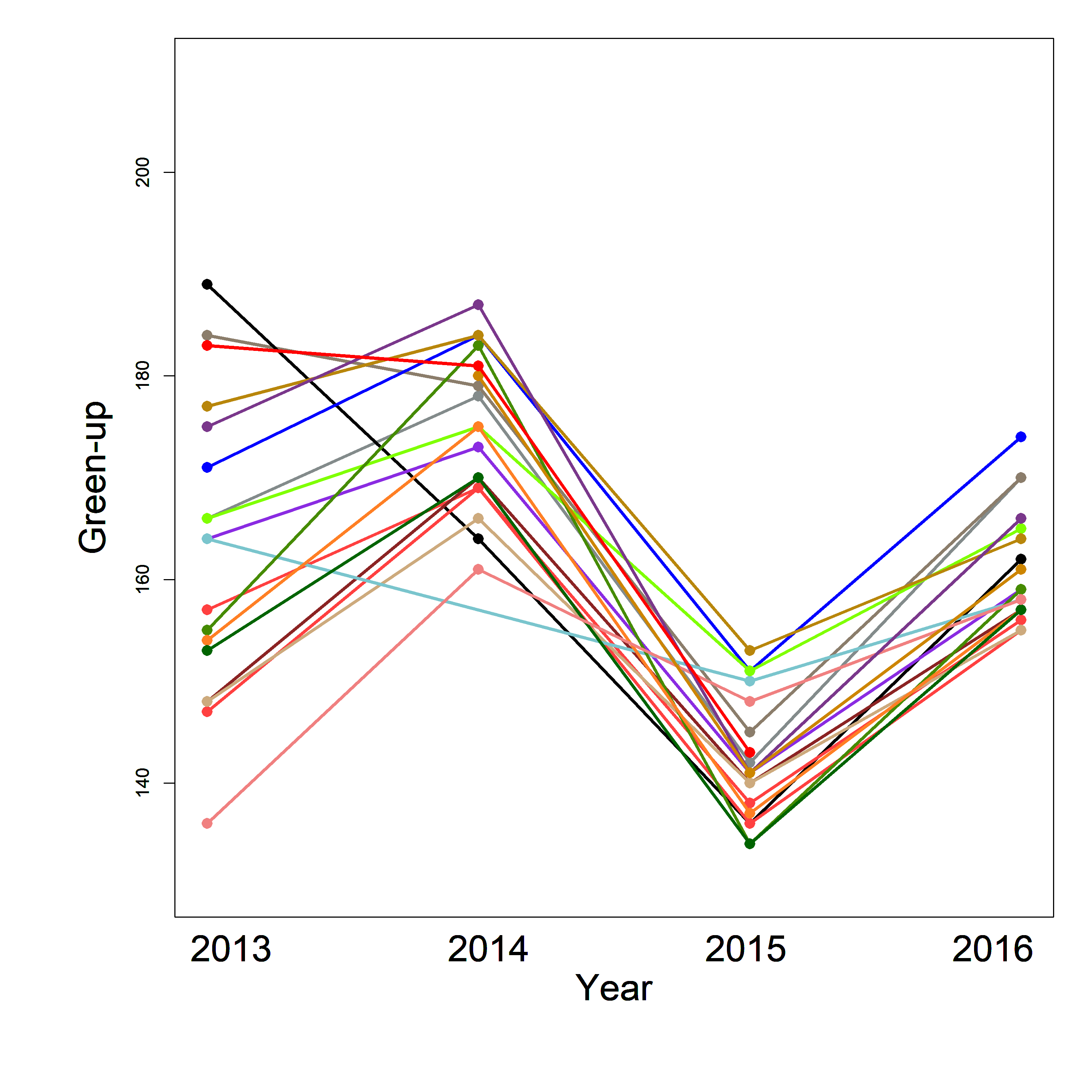

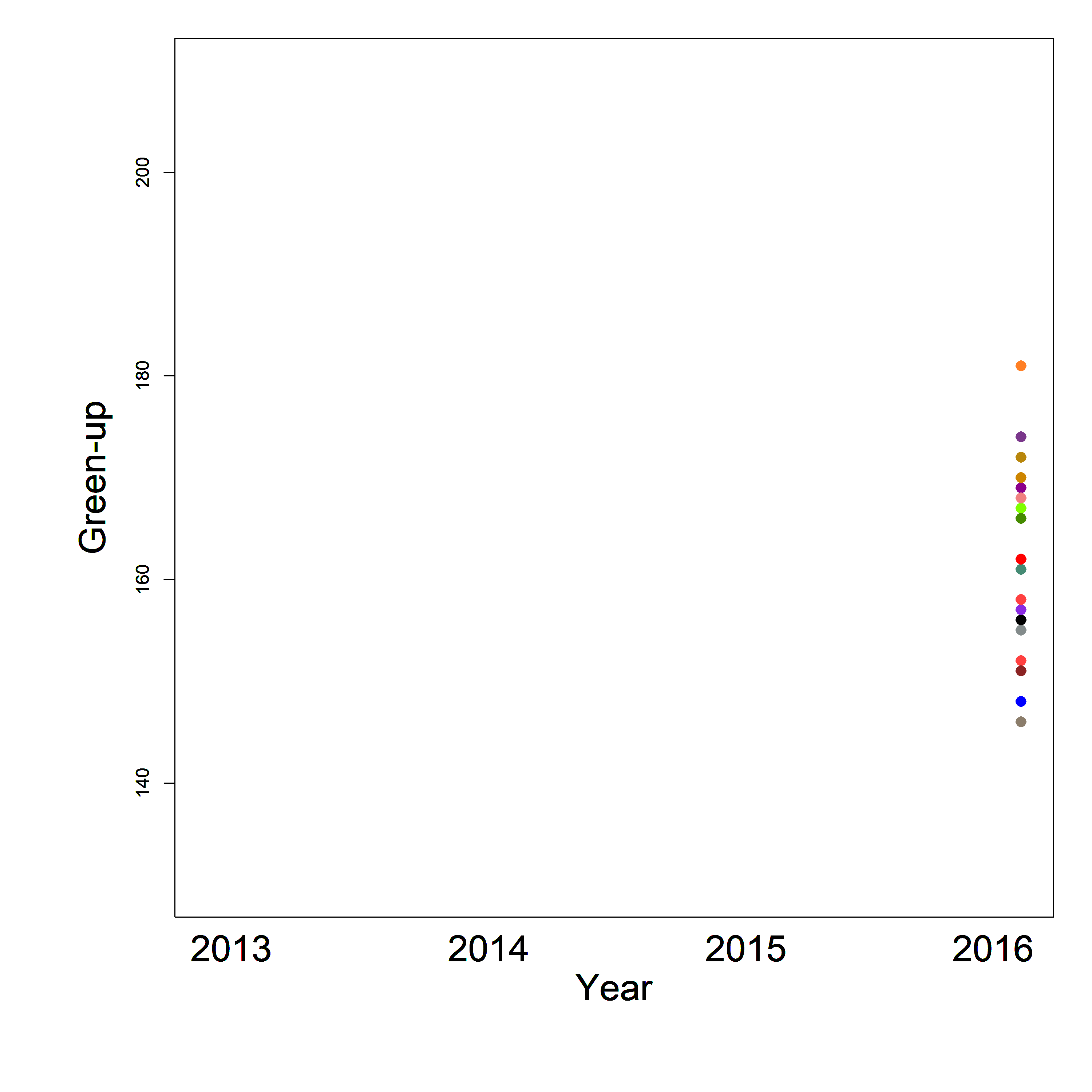

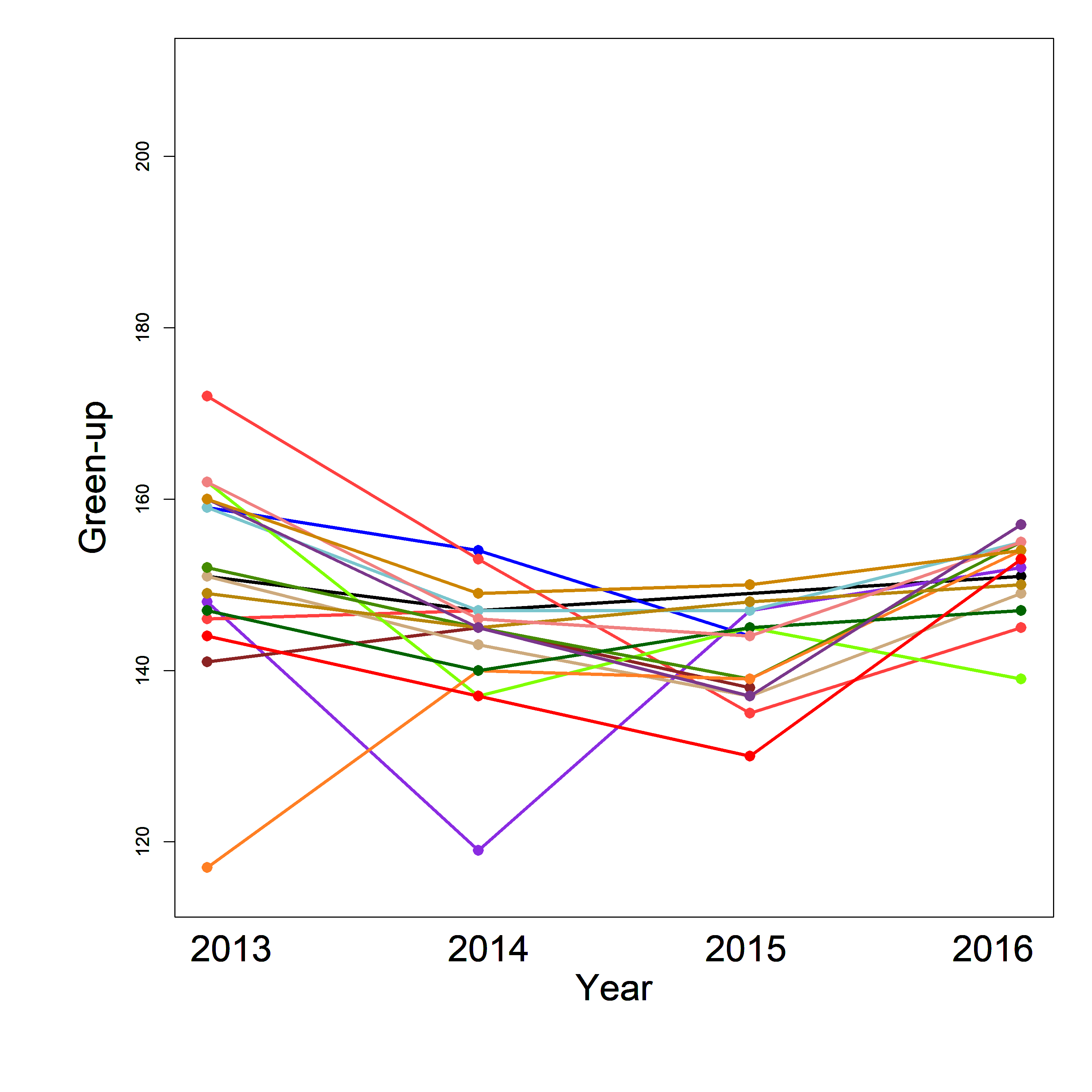

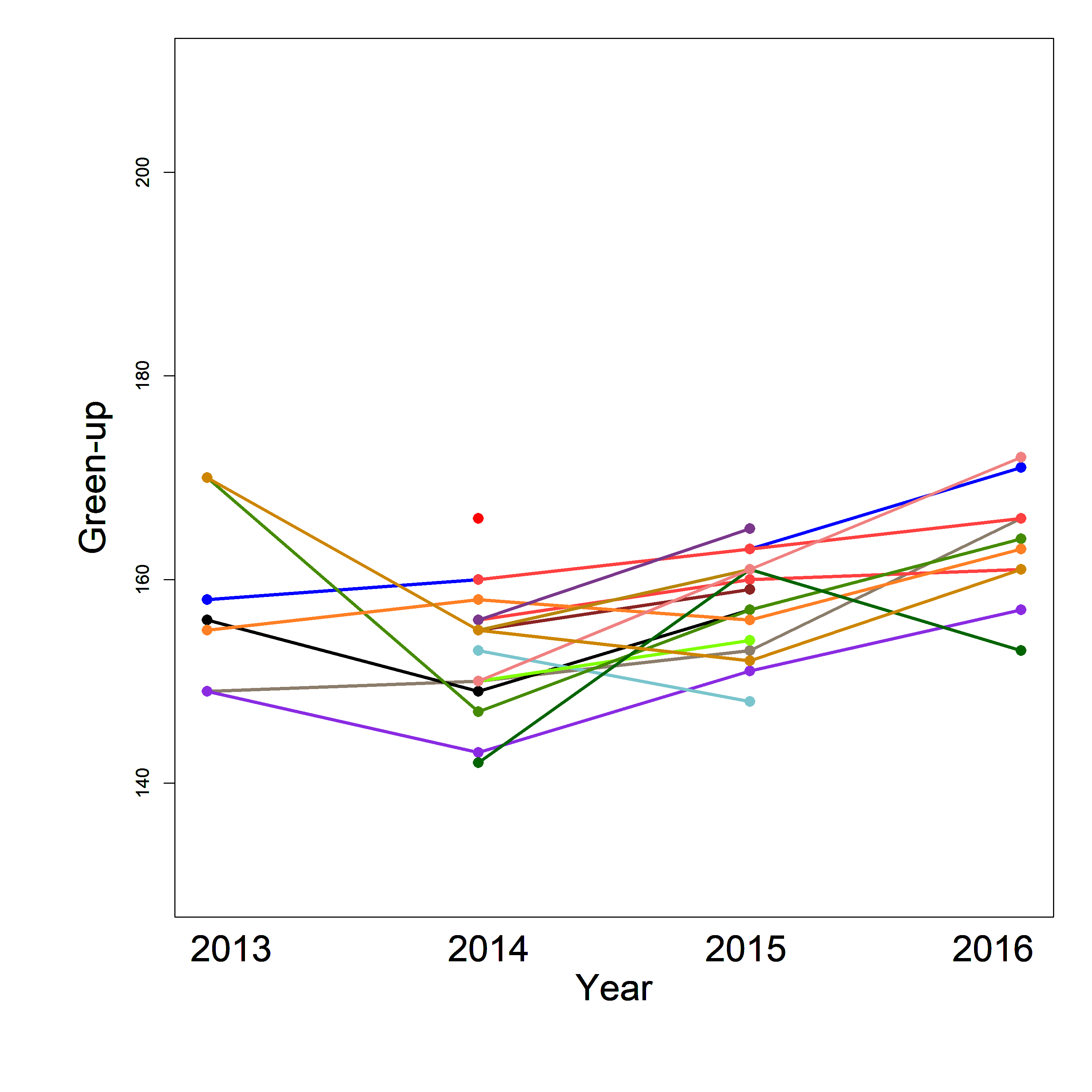

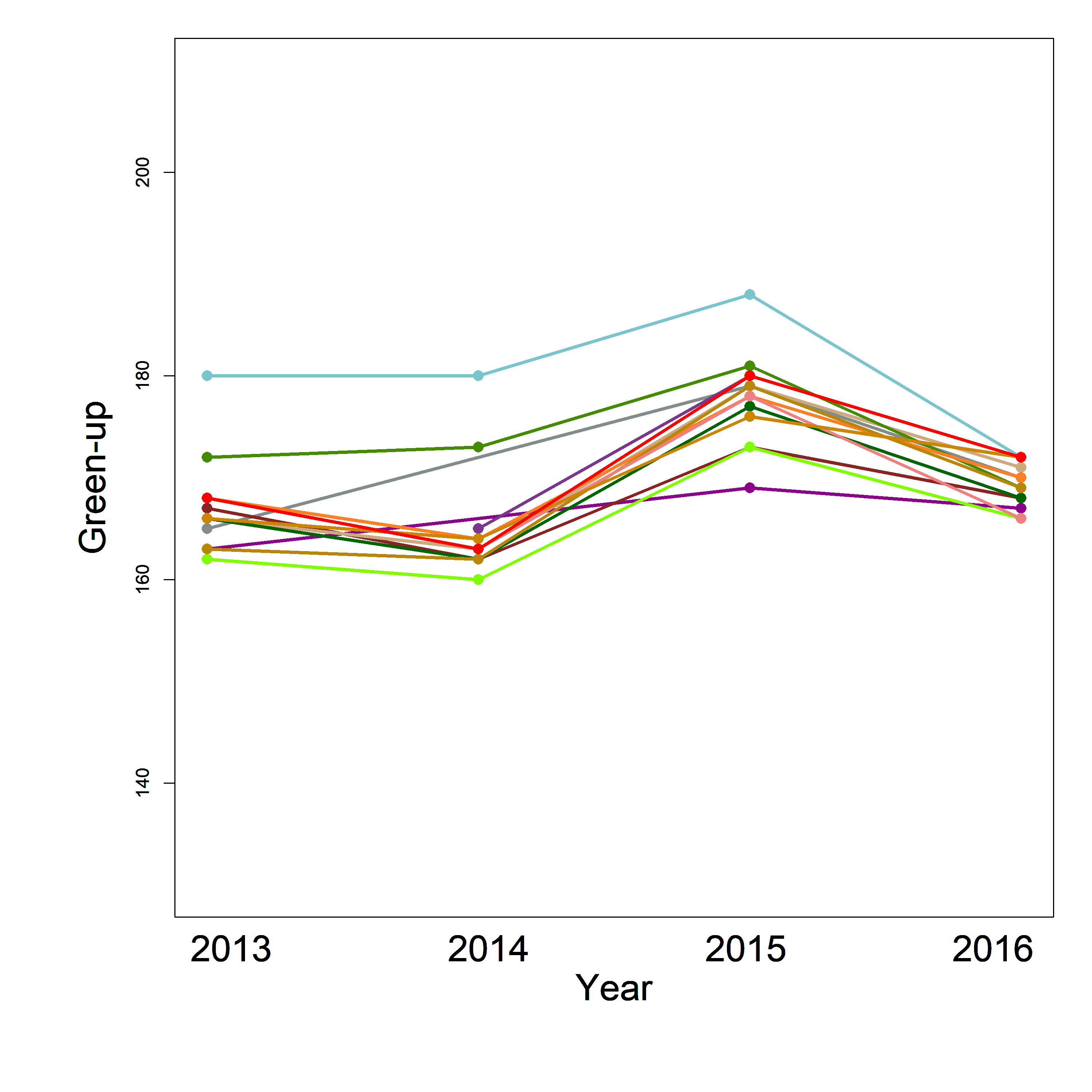


Figure S3. Interannual trends of the green-up phenology of 1) Temperate wet meadows (SCIRBI), 2) fluvial marshes (Maskinongé), 3)Peatlands (Lac-à-la-Tortue), 4) Peatlands (Bog-à-lanières) and 5) Arctic wet meadows (Umiujaq). Each color represents a unique plant community within each of the five landscapes. Each dot represents the onset of a particular community in a given year and each line represents the interannual trend of green-down date.


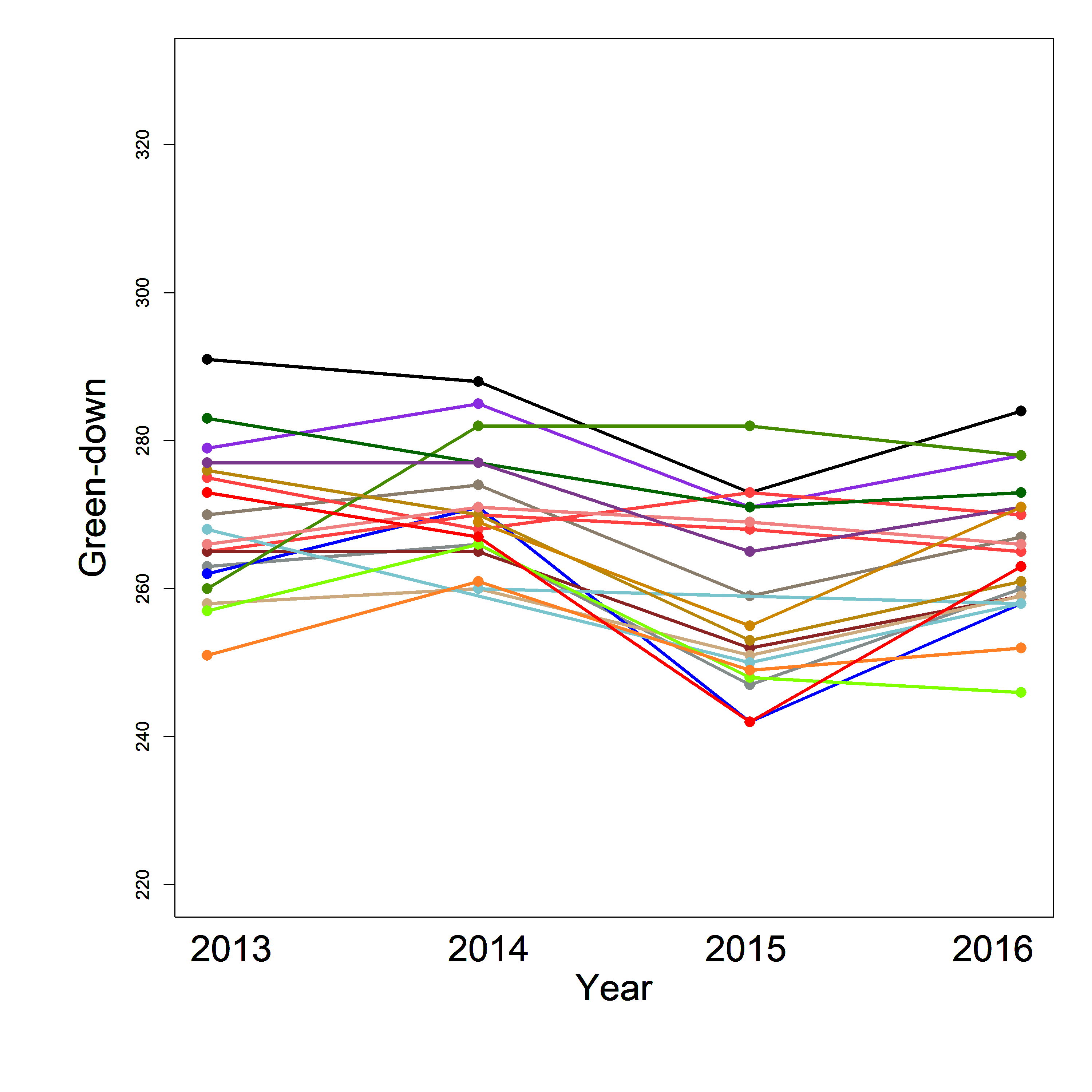

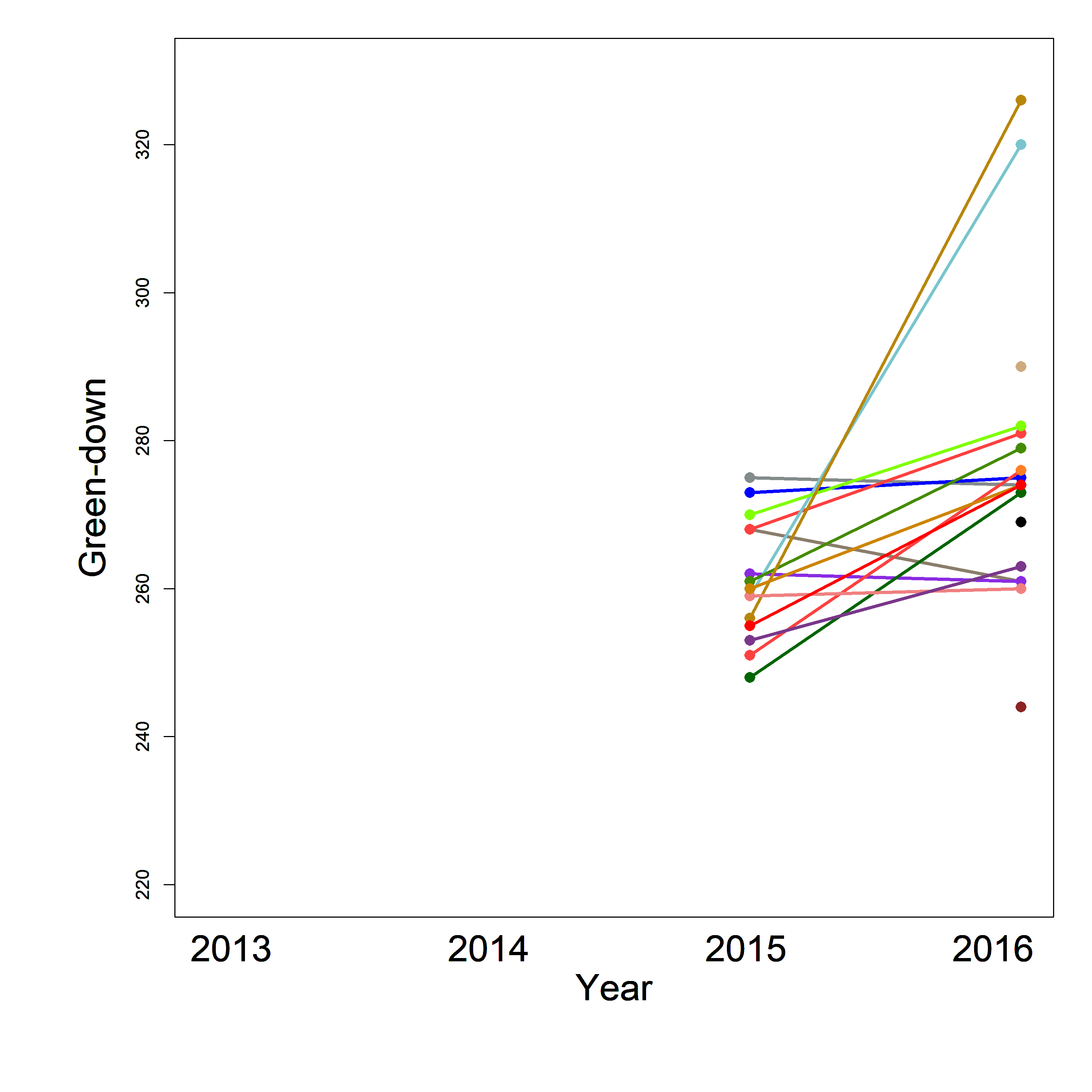

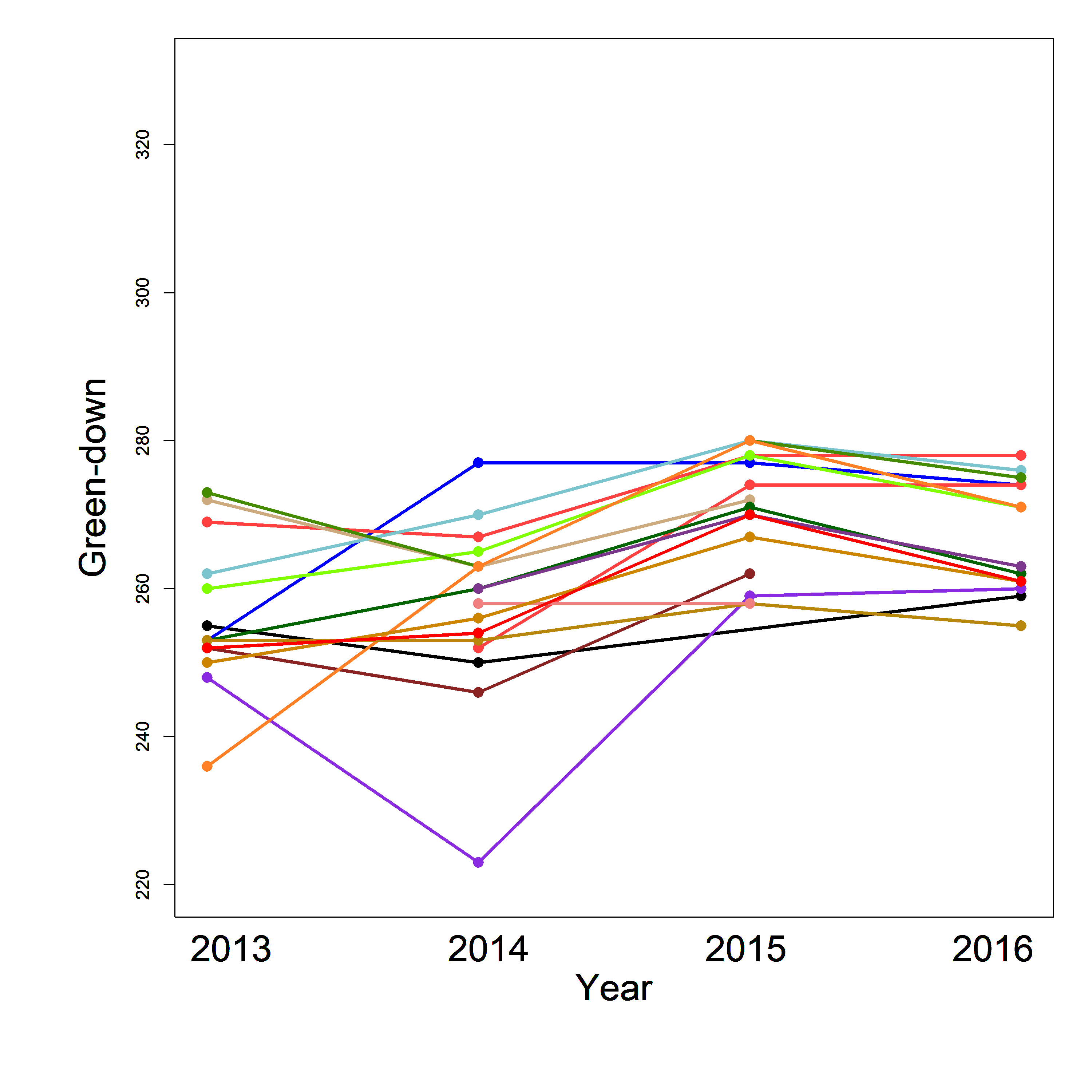

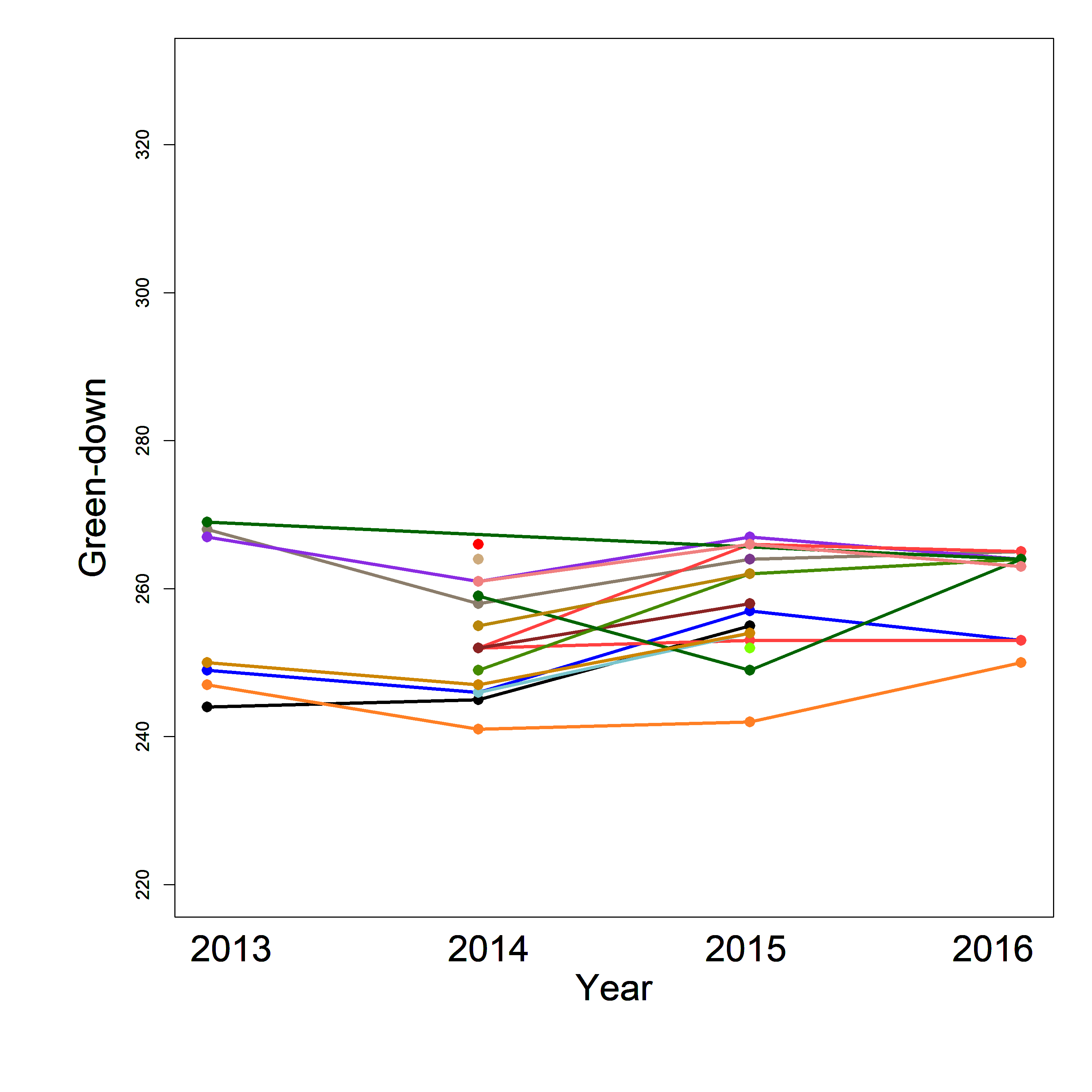

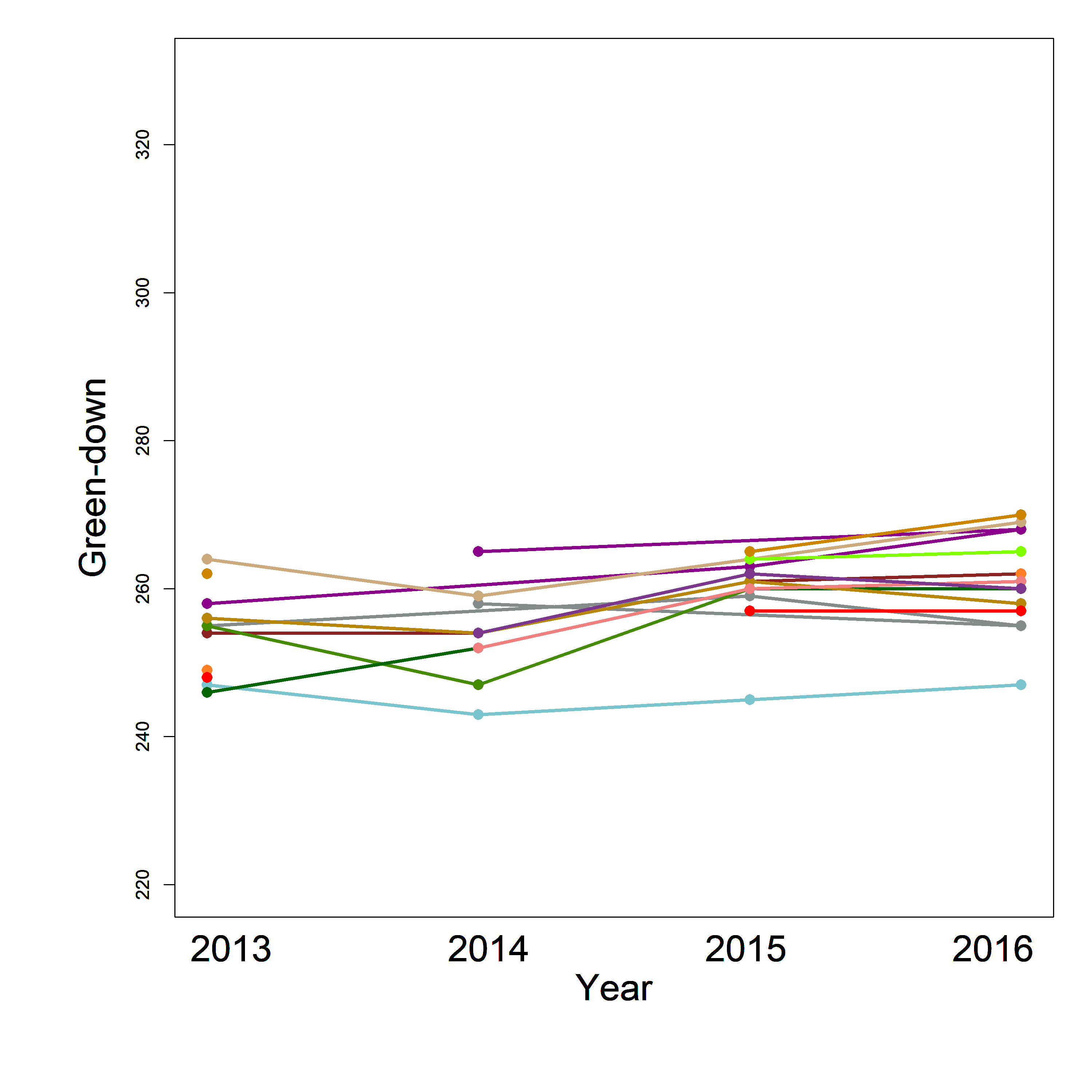


A)

A)

Figure S4. Interannual trends of the green-down phenology of 1) Temperate wet meadows (SCIRBI), 2) fluvial marshes (Maskinongé), 3)Peatlands (Lac-à-la-Tortue), 4) Peatlands (Bog-à-lanières) and 5) Arctic wet meadows (Umiujaq). Each color represents a unique plant community within each of the five landscapes. Each dot represents the offset of a particular community in a given year and each line represents the interannual trend of green-down date.


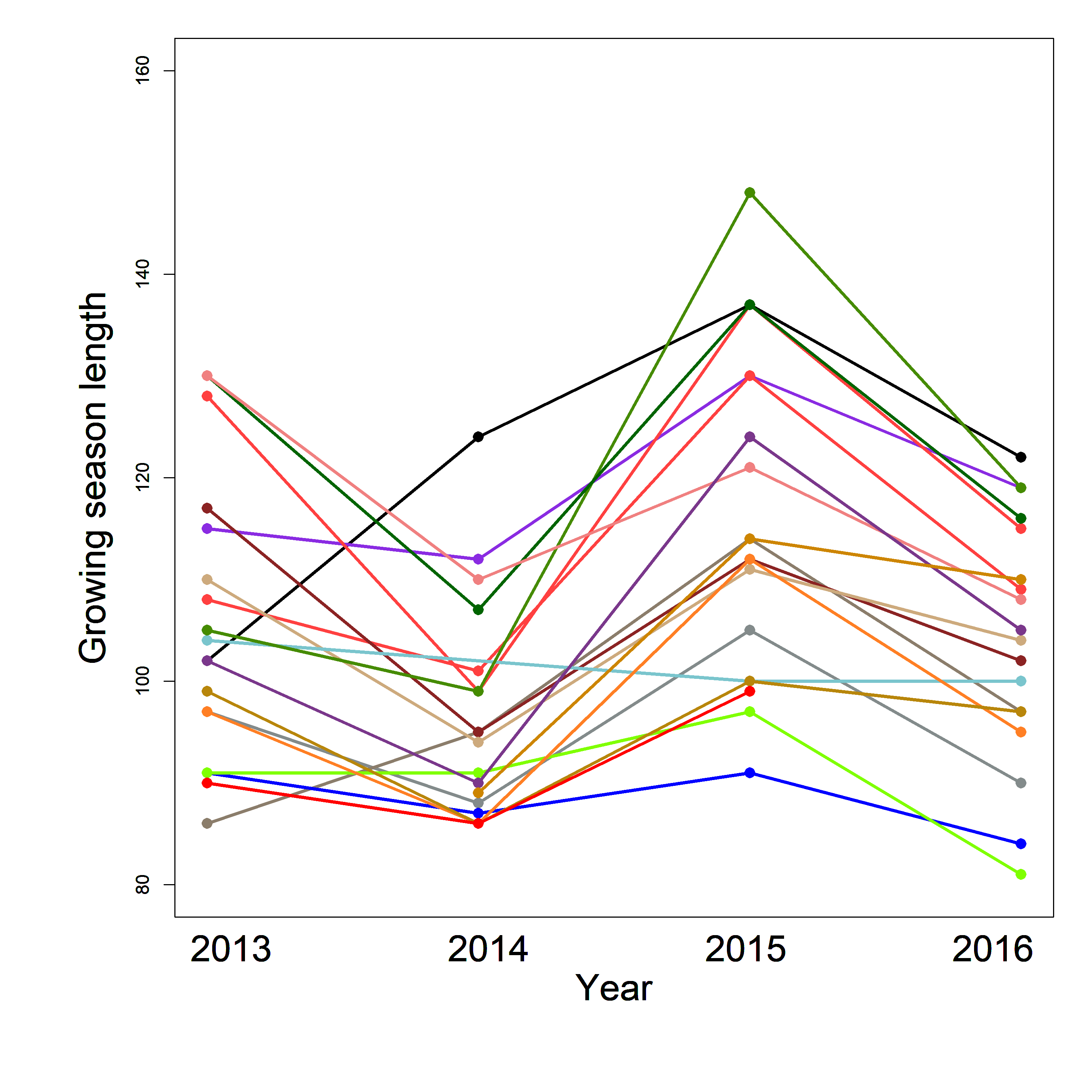

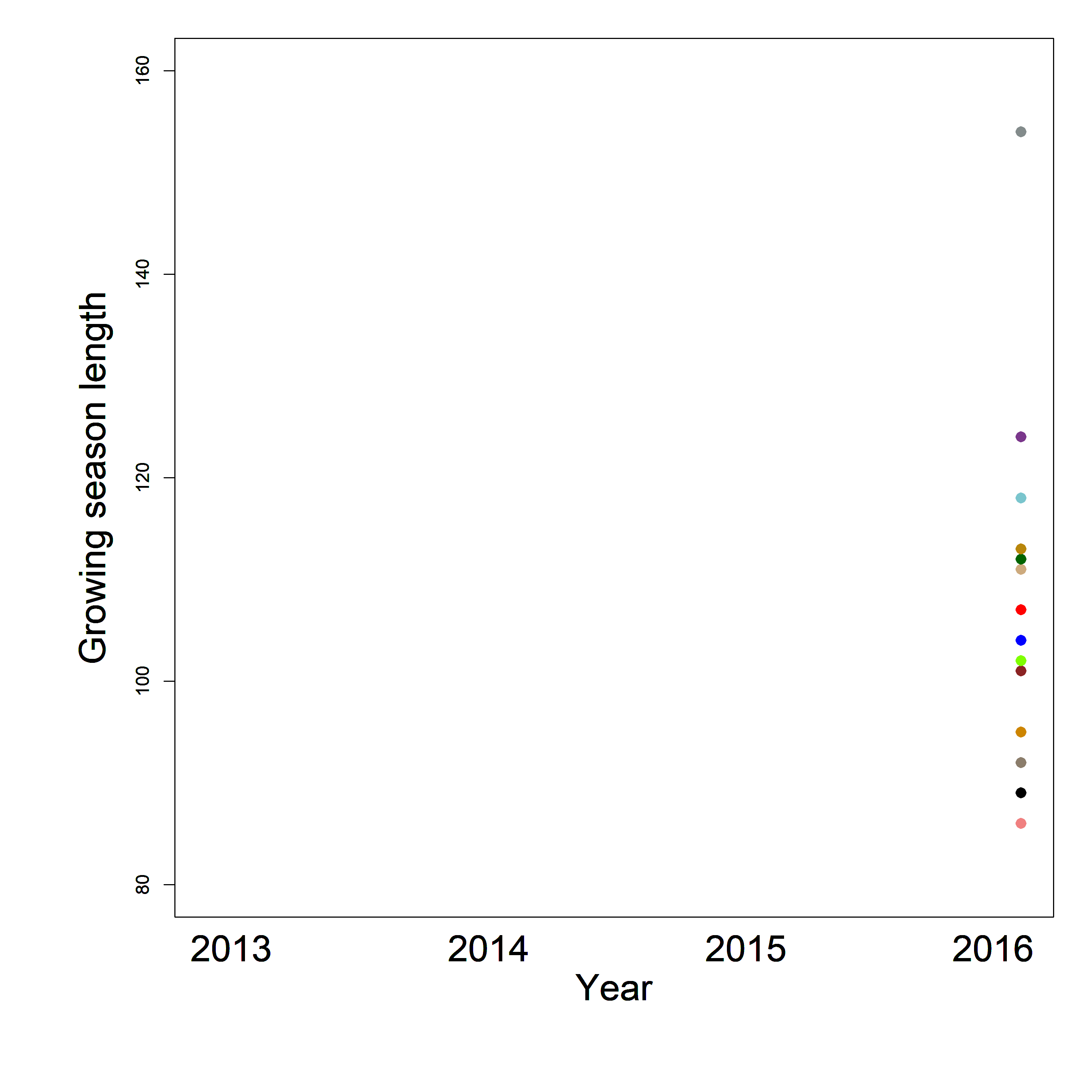

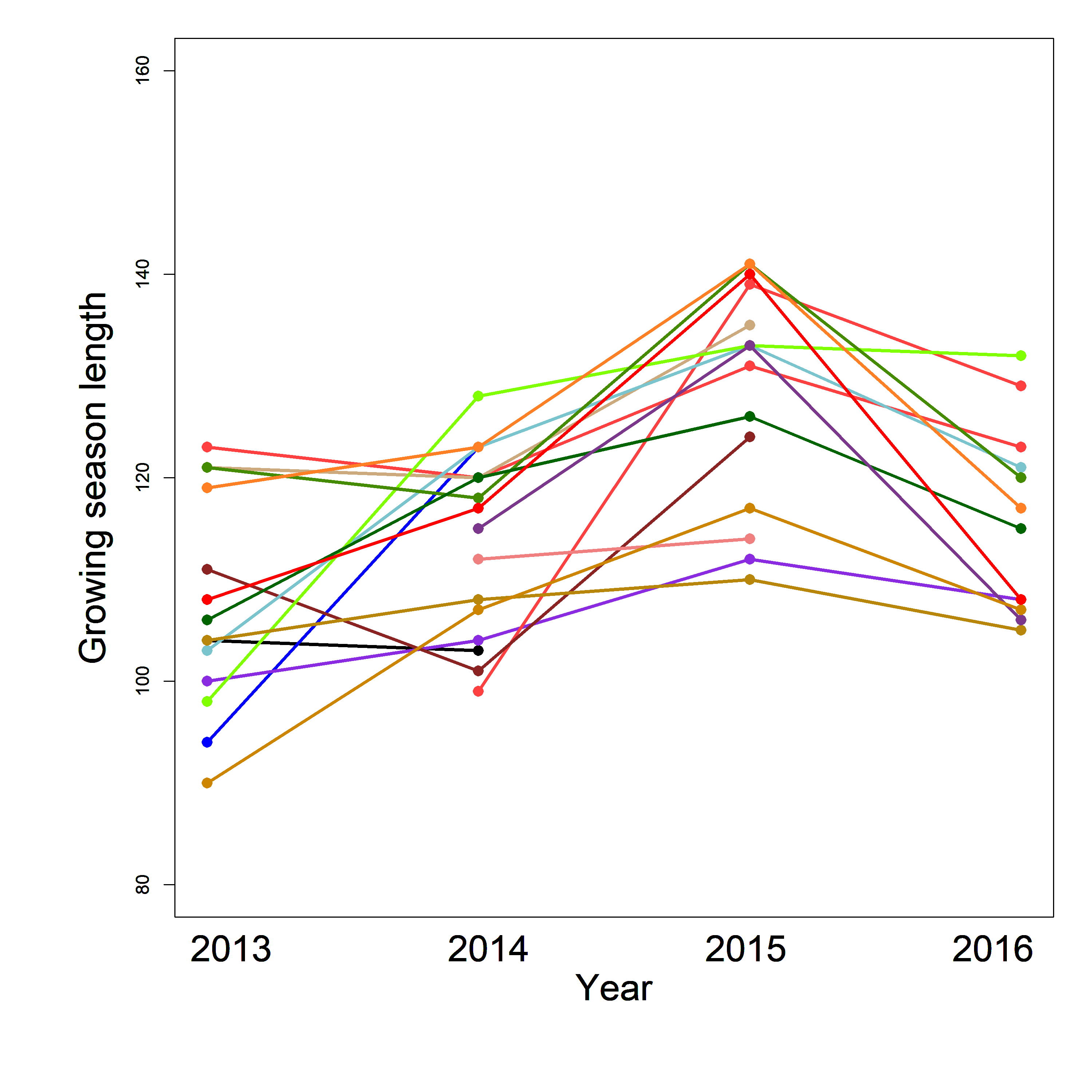


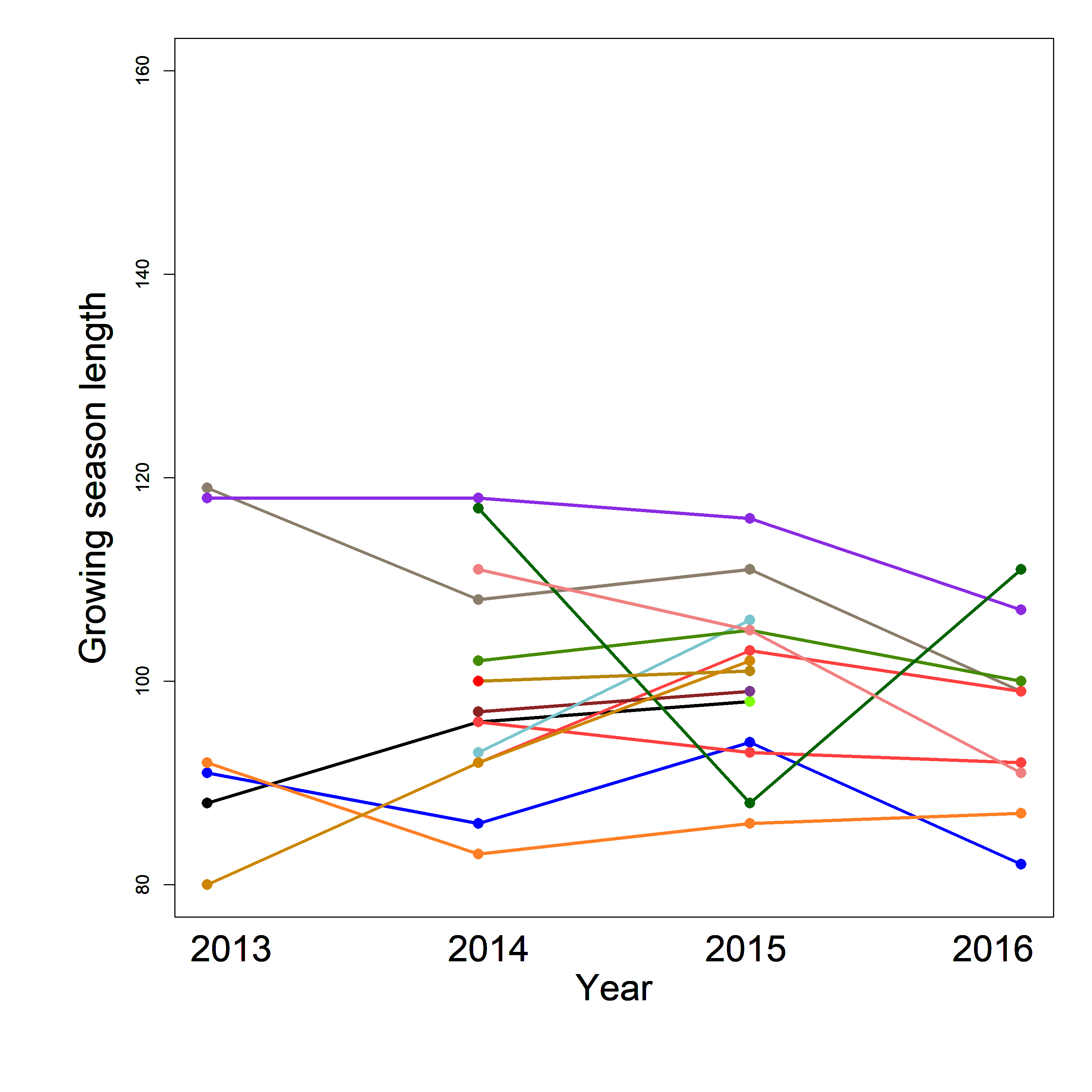

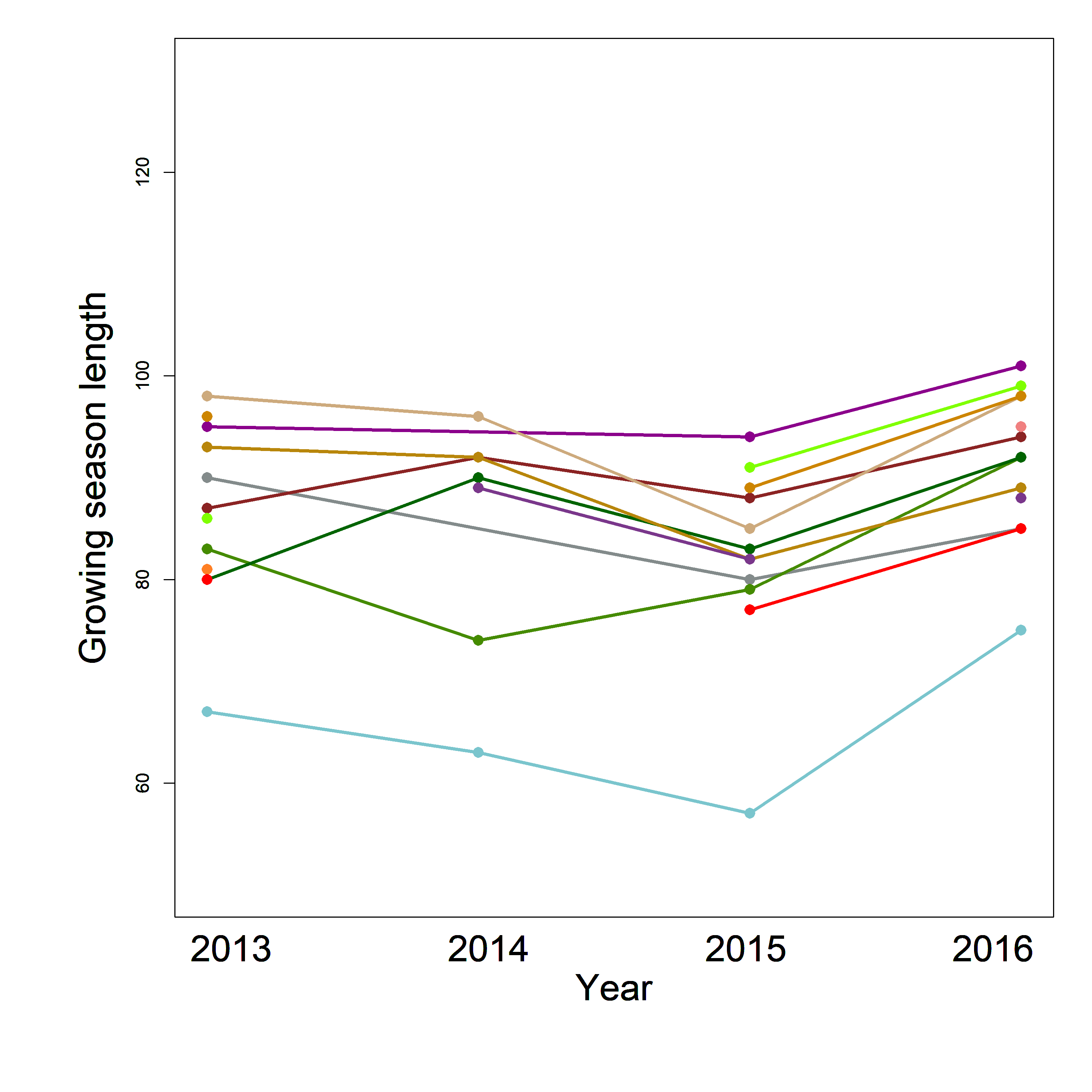


Figure S5. Interannual trends of the growing season length of 1) Temperate wet meadows (SCIRBI), 2) fluvial marshes (Maskinongé), 3)Peatlands (Lac-à-la-Tortue), 4) Peatlands (Bog-à-lanières) and 5) Arctic wet meadows (Umiujaq). Each color represents a unique plant community within each of the five landscapes. Each dot represents the phenology of a particular community in a given year and each line represents the interannual trend of growing season length.

Hierarchical modelling implementation using lm function within R environment

Example using Green.down

Factor1<- lm(pheno_off$Green.down~ pheno_off$Landscape)

resFactor2<- resid(Factor1)

Factor2<-lm(resFactor2~pheno_off$Species.richness)

resFactor2<-resid(Factor2)

Factor3<- lm(resFactor2~pheno_off$Landscape/(pheno_off$Year))

Factor4<- lm(resFactor2~pheno_off$Landscape(pheno_off$Year+pheno_off$Community.identity))

lm(resFactor2~pheno_off$Landscape/(pheno_off$Year+pheno_off$Community.identity + Year * Community.identity))

Landscape identity<- summary(Factor1)$adj.r.squared

Species richness<- ((1-Landscape identity)*summary(Factor2)$adj.r.squared)

Community temporal variance<- ((1 – Landscape identity- Species richness)*summary(Factor3)$adj.r.squared)

Community average functioning<- ((1-Landscape identity – Species richness)*(summary(Factor4)$adj.r.squared-summary(Factor3)$adj.r.squared))

Community asynchrony<- ((1-Landscape identity – Species richness)*(summary(Factor5-summary(Factor4)$adj.r.squared-summary(Factor3)$adj.r.squared))

Table S3. Complete database.

| Year | Landscape | Community identity | Green-up | Green-down | Growing season length | Species richness | Soil pH | Soil Moisture |
| --- | --- | --- | --- | --- | --- | --- | --- | --- |
| 2013 | scirbi | 21 | 175 | 277 | 102 | 6 | NA | NA |
| 2013 | scirbi | 23 | 183 | 273 | 90 | 6.25 | NA | NA |
| 2013 | scirbi | 24 | 166 | 263 | 97 | 6.25 | NA | NA |
| 2013 | scirbi | 25 | 184 | 270 | 86 | 5.75 | NA | NA |
| 2013 | scirbi | 26 | 189 | 291 | 102 | 2.75 | NA | NA |
| 2013 | scirbi | 27 | 171 | 262 | 91 | 5.25 | NA | NA |
| 2013 | scirbi | 29 | 164 | 279 | 115 | 4.75 | NA | NA |
| 2013 | scirbi | 30 | 147 | 275 | 128 | 2.25 | NA | NA |
| 2013 | scirbi | 31 | 157 | 265 | 108 | 3.25 | NA | NA |
| 2013 | scirbi | 32 | 148 | 265 | 117 | 4.25 | NA | NA |
| 2013 | scirbi | 33 | 148 | 258 | 110 | 3.25 | NA | NA |
| 2013 | scirbi | 34 | 164 | 268 | 104 | 5.5 | NA | NA |
| 2013 | scirbi | 35 | 166 | 257 | 91 | 3.75 | NA | NA |
| 2013 | scirbi | 36 | 155 | 260 | 105 | 1.25 | NA | NA |
| 2013 | scirbi | 37 | 154 | 251 | 97 | 2 | NA | NA |
| 2013 | scirbi | 38 | 153 | 283 | 130 | 1.25 | NA | NA |
| 2013 | scirbi | 39 | 177 | 276 | 99 | 4.25 | NA | NA |
| 2013 | scirbi | 40 | 136 | 266 | 130 | 5.5 | NA | NA |
| 2013 | tortue | 1 | 162 | NA | NA | 5 | NA | NA |
| 2013 | tortue | 10 | 160 | NA | NA | 5.75 | NA | NA |
| 2013 | tortue | 11 | 160 | 250 | 90 | 10 | NA | NA |
| 2013 | tortue | 12 | 144 | 252 | 108 | 10 | NA | NA |
| 2013 | tortue | 13 | 151 | 255 | 104 | 7.5 | NA | NA |
| 2013 | tortue | 15 | 159 | 253 | 94 | 7 | NA | NA |
| 2013 | tortue | 16 | 148 | 248 | 100 | 3.25 | NA | NA |
| 2013 | tortue | 17 | 172 | NA | NA | 5.25 | NA | NA |
| 2013 | tortue | 18 | 146 | 269 | 123 | 4.75 | NA | NA |
| 2013 | tortue | 19 | 141 | 252 | 111 | 7.25 | NA | NA |
| 2013 | tortue | 2 | 151 | 272 | 121 | 6 | NA | NA |
| 2013 | tortue | 20 | 159 | 262 | 103 | 7 | NA | NA |
| 2013 | tortue | 4 | 162 | 260 | 98 | 3.75 | NA | NA |
| 2013 | tortue | 5 | 152 | 273 | 121 | 3 | NA | NA |
| 2013 | tortue | 6 | 117 | 236 | 119 | 4 | NA | NA |
| 2013 | tortue | 7 | 147 | 253 | 106 | 4.25 | NA | NA |
| 2013 | tortue | 9 | 149 | 253 | 104 | 4 | NA | NA |
| 2013 | umiujaq | 61 | 163 | 258 | 95 | 1 | NA | NA |
| 2013 | umiujaq | 62 | 162 | 248 | 86 | 1 | NA | NA |
| 2013 | umiujaq | 63 | 165 | 255 | 90 | 3.75 | NA | NA |
| 2013 | umiujaq | 64 | 172 | 255 | 83 | 6.5 | NA | NA |
| 2013 | umiujaq | 65 | 168 | 249 | 81 | 5.75 | NA | NA |
| 2013 | umiujaq | 66 | 166 | 246 | 80 | 6.5 | NA | NA |
| 2013 | umiujaq | 67 | 163 | 256 | 93 | 4 | NA | NA |
| 2013 | umiujaq | 70 | 166 | 262 | 96 | 5.75 | NA | NA |
| 2013 | umiujaq | 71 | 168 | 248 | 80 | 3.5 | NA | NA |
| 2013 | umiujaq | 72 | 167 | 254 | 87 | 4 | NA | NA |
| 2013 | umiujaq | 73 | 166 | 264 | 98 | 2.25 | NA | NA |
| 2013 | umiujaq | 74 | 180 | 247 | 67 | 2.25 | NA | NA |
| 2013 | bog | 48 | 170 | NA | NA | 7.75 | NA | NA |
| 2013 | bog | 49 | 155 | 247 | 92 | 4 | NA | NA |
| 2013 | bog | 54 | 170 | 250 | 80 | 5.5 | NA | NA |
| 2013 | bog | 57 | 149 | 268 | 119 | 6.75 | NA | NA |
| 2013 | bog | 58 | 156 | 244 | 88 | 4 | NA | NA |
| 2013 | bog | 59 | 158 | 249 | 91 | 5.5 | NA | NA |
| 2013 | bog | 60 | 149 | 267 | 118 | 8.25 | NA | NA |
| 2014 | scirbi | 21 | 187 | 277 | 90 | 8.75 | NA | NA |
| 2014 | scirbi | 22 | 180 | 269 | 89 | 7 | NA | NA |
| 2014 | scirbi | 23 | 181 | 267 | 86 | 6.5 | NA | NA |
| 2014 | scirbi | 24 | 178 | 266 | 88 | 6 | NA | NA |
| 2014 | scirbi | 25 | 179 | 274 | 95 | 7.5 | NA | NA |
| 2014 | scirbi | 26 | 164 | 288 | 124 | 2.5 | NA | NA |
| 2014 | scirbi | 27 | 184 | 271 | 87 | 4.25 | NA | NA |
| 2014 | scirbi | 28 | 177 | 266 | 89 | 2.75 | NA | NA |
| 2014 | scirbi | 29 | 173 | 285 | 112 | 7.5 | NA | NA |
| 2014 | scirbi | 30 | 169 | 268 | 99 | 3.75 | NA | NA |
| 2014 | scirbi | 31 | 169 | 270 | 101 | 2.5 | NA | NA |
| 2014 | scirbi | 32 | 170 | 265 | 95 | 3 | NA | NA |
| 2014 | scirbi | 33 | 166 | 260 | 94 | 2 | NA | NA |
| 2014 | scirbi | 35 | 175 | 266 | 91 | 3.25 | NA | NA |
| 2014 | scirbi | 36 | 183 | 282 | 99 | 3 | NA | NA |
| 2014 | scirbi | 37 | 175 | 261 | 86 | 2.25 | NA | NA |
| 2014 | scirbi | 38 | 170 | 277 | 107 | 1.75 | NA | NA |
| 2014 | scirbi | 39 | 184 | 270 | 86 | 5.25 | NA | NA |
| 2014 | scirbi | 40 | 161 | 271 | 110 | 6 | NA | NA |
| 2014 | tortue | 1 | 146 | 258 | 112 | 5.75 | NA | NA |
| 2014 | tortue | 10 | 145 | 260 | 115 | 6.5 | NA | NA |
| 2014 | tortue | 11 | 149 | 256 | 107 | 8.25 | NA | NA |
| 2014 | tortue | 12 | 137 | 254 | 117 | 10.25 | NA | NA |
| 2014 | tortue | 13 | 147 | 250 | 103 | 7 | NA | NA |
| 2014 | tortue | 14 | 143 | 262 | 119 | 5 | NA | NA |
| 2014 | tortue | 15 | 154 | 277 | 123 | 7 | NA | NA |
| 2014 | tortue | 16 | 119 | 223 | 104 | 4.5 | NA | NA |
| 2014 | tortue | 17 | 153 | 252 | 99 | 5.75 | NA | NA |
| 2014 | tortue | 18 | 147 | 267 | 120 | 7 | NA | NA |
| 2014 | tortue | 19 | 145 | 246 | 101 | 6.5 | NA | NA |
| 2014 | tortue | 2 | 143 | 263 | 120 | 5.5 | NA | NA |
| 2014 | tortue | 20 | 147 | 270 | 123 | 7.75 | NA | NA |
| 2014 | tortue | 4 | 137 | 265 | 128 | 4 | NA | NA |
| 2014 | tortue | 5 | 145 | 263 | 118 | 4 | NA | NA |
| 2014 | tortue | 6 | 140 | 263 | 123 | 4.5 | NA | NA |
| 2014 | tortue | 7 | 140 | 260 | 120 | 4.25 | NA | NA |
| 2014 | tortue | 8 | 151 | 259 | 108 | 5 | NA | NA |
| 2014 | tortue | 9 | 145 | 253 | 108 | 4.5 | NA | NA |
| 2014 | bog | 41 | 156 | 252 | 96 | 6 | NA | NA |
| 2014 | bog | 42 | 160 | 252 | 92 | 7 | NA | NA |
| 2014 | bog | 43 | 155 | 252 | 97 | 7 | NA | NA |
| 2014 | bog | 44 | 147 | 264 | 117 | 7 | NA | NA |
| 2014 | bog | 45 | 153 | 246 | 93 | 4.75 | NA | NA |
| 2014 | bog | 46 | 150 | NA | NA | 8.75 | NA | NA |
| 2014 | bog | 48 | 147 | 249 | 102 | 6.25 | NA | NA |
| 2014 | bog | 49 | 158 | 241 | 83 | 4 | NA | NA |
| 2014 | bog | 50 | 142 | 259 | 117 | 7.25 | NA | NA |
| 2014 | bog | 51 | 155 | 255 | 100 | 8.75 | NA | NA |
| 2014 | bog | 52 | 150 | 261 | 111 | 7 | NA | NA |
| 2014 | bog | 53 | 156 | NA | NA | 10.5 | NA | NA |
| 2014 | bog | 54 | 155 | 247 | 92 | 3 | NA | NA |
| 2014 | bog | 555 | 166 | 266 | 100 | 8.75 | NA | NA |
| 2014 | bog | 556 | 158 | 267 | 109 | 5 | NA | NA |
| 2014 | bog | 57 | 150 | 258 | 108 | 7.25 | NA | NA |
| 2014 | bog | 58 | 149 | 245 | 96 | 5 | NA | NA |
| 2014 | bog | 59 | 160 | 246 | 86 | 4.25 | NA | NA |
| 2014 | bog | 60 | 143 | 261 | 118 | 6.75 | NA | NA |
| 2014 | umiujaq | 62 | 160 | NA | NA | 1 | NA | NA |
| 2014 | umiujaq | 64 | 173 | 247 | 74 | 7 | NA | NA |
| 2014 | umiujaq | 65 | 164 | NA | NA | 6 | NA | NA |
| 2014 | umiujaq | 66 | 162 | 252 | 90 | 7 | NA | NA |
| 2014 | umiujaq | 67 | 162 | 254 | 92 | 5 | NA | NA |
| 2014 | umiujaq | 68 | 163 | 252 | 89 | 5 | NA | NA |
| 2014 | umiujaq | 69 | 165 | 254 | 89 | 5 | NA | NA |
| 2014 | umiujaq | 70 | 164 | NA | NA | 5.75 | NA | NA |
| 2014 | umiujaq | 71 | 163 | NA | NA | 3.5 | NA | NA |
| 2014 | umiujaq | 72 | 162 | 254 | 92 | 4.66 | NA | NA |
| 2014 | umiujaq | 73 | 163 | 259 | 96 | 2 | NA | NA |
| 2014 | umiujaq | 74 | 180 | 243 | 63 | 3.66 | NA | NA |
| 2015 | scirbi | 21 | 141 | 265 | 124 | 9.75 | 5.78 | 46.575 |
| 2015 | scirbi | 22 | 141 | 255 | 114 | 9.25 | 4.97 | 53.9 |
| 2015 | scirbi | 23 | 143 | 242 | 99 | 6 | 5.51 | 50.1 |
| 2015 | scirbi | 24 | 142 | 247 | 105 | 8 | 5.345 | 55.65 |
| 2015 | scirbi | 25 | 145 | 259 | 114 | 6.75 | 5.955 | 45.425 |
| 2015 | scirbi | 26 | 136 | 273 | 137 | 3.25 | 5.69 | 22.55 |
| 2015 | scirbi | 27 | 151 | 242 | 91 | 6 | 5.295 | 55.3 |
| 2015 | scirbi | 28 | 140 | 259 | 119 | 2.75 | 5.715 | 35.475 |
| 2015 | scirbi | 29 | 141 | 271 | 130 | 7.5 | 5.435 | 22.35 |
| 2015 | scirbi | 30 | 136 | 273 | 137 | 3.5 | 5.335 | 22.675 |
| 2015 | scirbi | 31 | 138 | 268 | 130 | 2.75 | 5.26 | 27.725 |
| 2015 | scirbi | 32 | 140 | 252 | 112 | 3.75 | 5.415 | 21.625 |
| 2015 | scirbi | 33 | 140 | 251 | 111 | 2.25 | 5.75 | 14.225 |
| 2015 | scirbi | 34 | 150 | 250 | 100 | 10.25 | 5.19 | 38.575 |
| 2015 | scirbi | 35 | 151 | 248 | 97 | 3.25 | 5.1 | 52.575 |
| 2015 | scirbi | 36 | 134 | 282 | 148 | 2.5 | 5.645 | 43.75 |
| 2015 | scirbi | 37 | 137 | 249 | 112 | 2.75 | 5.755 | 36.975 |
| 2015 | scirbi | 38 | 134 | 271 | 137 | 3 | 5.72 | 34.575 |
| 2015 | scirbi | 39 | 153 | 253 | 100 | 6.25 | 5.255 | 51.325 |
| 2015 | scirbi | 40 | 148 | 269 | 121 | 7.5 | 5.475 | 57.05 |
| 2015 | tortue | 1 | 144 | 258 | 114 | 4.5 | 3.4975 | 88.3 |
| 2015 | tortue | 10 | 137 | 270 | 133 | 6 | 3.405 | 58.075 |
| 2015 | tortue | 11 | 150 | 267 | 117 | 9.25 | 3.845 | 80.075 |
| 2015 | tortue | 12 | 130 | 270 | 140 | 10 | 3.3925 | 70.575 |
| 2015 | tortue | 14 | 142 | 272 | 130 | 5 | 3.615 | 83.8 |
| 2015 | tortue | 15 | 144 | 277 | 133 | 7 | 3.3775 | 77.875 |
| 2015 | tortue | 16 | 147 | 259 | 112 | 3.5 | 3.465 | 82.5 |
| 2015 | tortue | 17 | 135 | 274 | 139 | 7.25 | 3.4425 | 77.125 |
| 2015 | tortue | 18 | 147 | 278 | 131 | 5 | 3.4375 | 66.225 |
| 2015 | tortue | 19 | 138 | 262 | 124 | 5.75 | 3.6325 | 83.25 |
| 2015 | tortue | 2 | 137 | 272 | 135 | 7 | 3.4675 | 86.65 |
| 2015 | tortue | 20 | 147 | 280 | 133 | 9.25 | 3.45 | 67.225 |
| 2015 | tortue | 3 | 148 | 264 | 116 | 1 | 3.41 | 87.775 |
| 2015 | tortue | 4 | 145 | 278 | 133 | 4 | 3.41 | 86 |
| 2015 | tortue | 5 | 139 | 280 | 141 | 4.5 | 3.49 | 83.15 |
| 2015 | tortue | 6 | 139 | 280 | 141 | 4 | 3.4125 | 45.8 |
| 2015 | tortue | 7 | 145 | 271 | 126 | 4 | 3.41 | 86 |
| 2015 | tortue | 8 | 152 | 275 | 123 | 5.5 | 3.4125 | 74.725 |
| 2015 | tortue | 9 | 148 | 258 | 110 | 4 | 3.355 | 85.3 |
| 2015 | bog | 41 | 160 | 253 | 93 | 7.5 | 3.995 | 72.825 |
| 2015 | bog | 42 | 163 | 266 | 103 | 6 | 3.9475 | 37.225 |
| 2015 | bog | 43 | 159 | 258 | 99 | 5.75 | 3.9225 | 59.45 |
| 2015 | bog | 45 | 148 | 254 | 106 | 3.25 | 4.26 | 77.35 |
| 2015 | bog | 46 | 154 | 252 | 98 | 8.25 | 4.25 | 63.225 |
| 2015 | bog | 48 | 157 | 262 | 105 | 5.75 | 4.0375 | 56.125 |
| 2015 | bog | 49 | 156 | 242 | 86 | 7 | 4.0975 | 73.075 |
| 2015 | bog | 50 | 161 | 249 | 88 | 8.25 | 3.9975 | 66.125 |
| 2015 | bog | 51 | 161 | 262 | 101 | 9.75 | 3.97 | 65.35 |
| 2015 | bog | 52 | 161 | 266 | 105 | 5.75 | 3.935 | 50.825 |
| 2015 | bog | 53 | 165 | 264 | 99 | 9 | 3.9775 | 37.6 |
| 2015 | bog | 54 | 152 | 254 | 102 | 4 | 4.115 | 72.2 |
| 2015 | bog | 55 | 158 | NA | NA | 4 | 3.9325 | 67.3 |
| 2015 | bog | 56 | 158 | 254 | 96 | 8.75 | 4.16 | 51.6 |
| 2015 | bog | 57 | 153 | 264 | 111 | 9 | 3.8325 | 63.075 |
| 2015 | bog | 58 | 157 | 255 | 98 | 5 | 3.8825 | 71.25 |
| 2015 | bog | 59 | 163 | 257 | 94 | 5.25 | 3.9725 | 62.6 |
| 2015 | bog | 60 | 151 | 267 | 116 | 8 | 3.9975 | 65.075 |
| 2015 | bog | 89 | 156 | 266 | 110 | 7.5 | NA | NA |
| 2015 | umiujaq | 61 | 169 | 263 | 94 | 1 | 5 | 21.725 |
| 2015 | umiujaq | 62 | 173 | 264 | 91 | 1 | 4.92 | 18.575 |
| 2015 | umiujaq | 63 | 179 | 259 | 80 | 3.75 | 4.7 | 72.95 |
| 2015 | umiujaq | 64 | 181 | 260 | 79 | 5.5 | 5.34 | 78.175 |
| 2015 | umiujaq | 65 | 178 | NA | NA | 4.25 | 4.68 | 51.45 |
| 2015 | umiujaq | 66 | 177 | 260 | 83 | 5.75 | 5.55 | 52 |
| 2015 | umiujaq | 67 | 179 | 261 | 82 | 5.75 | 5.21 | 35.65 |
| 2015 | umiujaq | 68 | 178 | 260 | 82 | 5 | 5.62 | 46.175 |
| 2015 | umiujaq | 69 | 180 | 262 | 82 | 5 | 5.71 | 50.9 |
| 2015 | umiujaq | 70 | 176 | 265 | 89 | 6 | 4.67 | 50.175 |
| 2015 | umiujaq | 71 | 180 | 257 | 77 | 5 | 5.86 | 48.075 |
| 2015 | umiujaq | 72 | 173 | 261 | 88 | 4 | 5.38 | 47.125 |
| 2015 | umiujaq | 73 | 179 | 264 | 85 | 2 | 5.03 | 43.7 |
| 2015 | umiujaq | 74 | 188 | 245 | 57 | 2.25 | 5.18 | 86.6 |
| 2015 | umiujaq | 79 | 188 | 258 | 70 | 2.5 | 5.77 | 75.075 |
| 2015 | umiujaq | 81 | 175 | 258 | 83 | 6.5 | 6 | 40.6 |
| 2015 | umiujaq | 82 | 171 | 262 | 91 | 2.5 | 5.32 | 25.275 |
| 2015 | umiujaq | 83 | 179 | 263 | 84 | 2.25 | 5.45 | 40.025 |
| 2015 | umiujaq | 85 | 183 | 261 | 78 | 4.25 | 5.7 | 79.625 |
| 2015 | umiujaq | 86 | 181 | 259 | 78 | 6.5 | 4.89 | 73.075 |
| 2015 | umiujaq | 87 | 186 | 258 | 72 | 3.5 | 5.59 | 78.775 |
| 2015 | umiujaq | 88 | 178 | 263 | 85 | 3 | 5.52 | 79.5 |
| 2016 | scirbi | 21 | 166 | 271 | 105 | 10.25 | NA | NA |
| 2016 | scirbi | 22 | 161 | 271 | 110 | 10.5 | NA | NA |
| 2016 | scirbi | 24 | 170 | 260 | 90 | 7 | NA | NA |
| 2016 | scirbi | 25 | 170 | 267 | 97 | 9.5 | NA | NA |
| 2016 | scirbi | 26 | 162 | 284 | 122 | 3.75 | NA | NA |
| 2016 | scirbi | 27 | 174 | 258 | 84 | 6 | NA | NA |
| 2016 | scirbi | 28 | 164 | NA | NA | 3.75 | NA | NA |
| 2016 | scirbi | 29 | 159 | 278 | 119 | 5.5 | NA | NA |
| 2016 | scirbi | 30 | 155 | 270 | 115 | 5 | NA | NA |
| 2016 | scirbi | 31 | 156 | 265 | 109 | 2.25 | NA | NA |
| 2016 | scirbi | 32 | 157 | 259 | 102 | 4.25 | NA | NA |
| 2016 | scirbi | 33 | 155 | 259 | 104 | 3 | NA | NA |
| 2016 | scirbi | 34 | 158 | 258 | 100 | 9.5 | NA | NA |
| 2016 | scirbi | 35 | 165 | 246 | 81 | 3.5 | NA | NA |
| 2016 | scirbi | 36 | 159 | 278 | 119 | 3.5 | NA | NA |
| 2016 | scirbi | 37 | 157 | 252 | 95 | 3.5 | NA | NA |
| 2016 | scirbi | 38 | 157 | 273 | 116 | 3.5 | NA | NA |
| 2016 | scirbi | 39 | 164 | 261 | 97 | 3.75 | NA | NA |
| 2016 | scirbi | 40 | 158 | 266 | 108 | 6.5 | NA | NA |
| 2016 | tortue | 1 | 155 | NA | NA | 6.25 | NA | NA |
| 2016 | tortue | 10 | 157 | 263 | 106 | 7.75 | NA | NA |
| 2016 | tortue | 11 | 154 | 261 | 107 | 9.25 | NA | NA |
| 2016 | tortue | 12 | 153 | 261 | 108 | 9.25 | NA | NA |
| 2016 | tortue | 13 | 151 | 259 | 108 | 7.75 | NA | NA |
| 2016 | tortue | 14 | 150 | NA | NA | 6 | NA | NA |
| 2016 | tortue | 16 | 152 | 260 | 108 | 4 | NA | NA |
| 2016 | tortue | 17 | 145 | 274 | 129 | 7.5 | NA | NA |
| 2016 | tortue | 18 | 155 | 278 | 123 | 6 | NA | NA |
| 2016 | tortue | 2 | 149 | NA | NA | 5 | NA | NA |
| 2016 | tortue | 20 | 155 | 276 | 121 | 7 | NA | NA |
| 2016 | tortue | 4 | 139 | 271 | 132 | 5 | NA | NA |
| 2016 | tortue | 5 | 155 | 275 | 120 | 5 | NA | NA |
| 2016 | tortue | 6 | 154 | 271 | 117 | 4 | NA | NA |
| 2016 | tortue | 7 | 147 | 262 | 115 | 5 | NA | NA |
| 2016 | tortue | 8 | 150 | 273 | 123 | 5 | NA | NA |
| 2016 | tortue | 9 | 150 | 255 | 105 | 5 | NA | NA |
| 2016 | bog | 41 | 161 | 253 | 92 | 7.75 | NA | NA |
| 2016 | bog | 42 | 166 | 265 | 99 | 8 | NA | NA |
| 2016 | bog | 47 | 153 | 257 | 104 | 9.75 | NA | NA |
| 2016 | bog | 48 | 164 | 264 | 100 | 7.5 | NA | NA |
| 2016 | bog | 49 | 163 | 250 | 87 | 6 | NA | NA |
| 2016 | bog | 50 | 153 | 264 | 111 | 10.75 | NA | NA |
| 2016 | bog | 52 | 172 | 263 | 91 | 6.5 | NA | NA |
| 2016 | bog | 54 | 161 | NA | NA | 5.75 | NA | NA |
| 2016 | bog | 55 | 168 | 242 | 74 | 5.5 | NA | NA |
| 2016 | bog | 57 | 166 | 265 | 99 | 5 | NA | NA |
| 2016 | bog | 59 | 171 | 253 | 82 | 5 | NA | NA |
| 2016 | bog | 60 | 157 | 264 | 107 | 7.25 | NA | NA |
| 2016 | umiujaq | 61 | 167 | 268 | 101 | 1 | NA | NA |
| 2016 | umiujaq | 62 | 166 | 265 | 99 | 1 | NA | NA |
| 2016 | umiujaq | 63 | 170 | 255 | 85 | 3 | NA | NA |
| 2016 | umiujaq | 64 | 169 | 261 | 92 | 5.5 | NA | NA |
| 2016 | umiujaq | 65 | 170 | 262 | 92 | 7.5 | NA | NA |
| 2016 | umiujaq | 66 | 168 | 260 | 92 | 6.25 | NA | NA |
| 2016 | umiujaq | 67 | 169 | 258 | 89 | 6 | NA | NA |
| 2016 | umiujaq | 68 | 166 | 261 | 95 | 4.25 | NA | NA |
| 2016 | umiujaq | 69 | 172 | 260 | 88 | 4.5 | NA | NA |
| 2016 | umiujaq | 70 | 172 | 270 | 98 | 6.25 | NA | NA |
| 2016 | umiujaq | 71 | 172 | 257 | 85 | 4.75 | NA | NA |
| 2016 | umiujaq | 72 | 168 | 262 | 94 | 4.5 | NA | NA |
| 2016 | umiujaq | 73 | 171 | 269 | 98 | 2.25 | NA | NA |
| 2016 | umiujaq | 74 | 172 | 247 | 75 | 3.5 | NA | NA |
| 2016 | umiujaq | 76 | 185 | 259 | 74 | 2.5 | NA | NA |
| 2016 | umiujaq | 77 | 172 | NA | NA | 7.75 | NA | NA |
| 2016 | umiujaq | 78 | 175 | 255 | 80 | 6.75 | NA | NA |
| 2016 | umiujaq | 79 | 182 | 255 | 73 | 3.75 | NA | NA |
| 2016 | umiujaq | 83 | 171 | NA | NA | 1 | NA | NA |
| 2016 | umiujaq | 84 | 171 | 252 | 81 | 3.75 | NA | NA |
| 2016 | umiujaq | 85 | 178 | 258 | 80 | 4.75 | NA | NA |
| 2016 | umiujaq | 86 | 173 | 258 | 85 | 7 | NA | NA |
| 2016 | umiujaq | 87 | 179 | 252 | 73 | 4.25 | NA | NA |
| 2016 | umiujaq | 88 | 172 | NA | NA | 1.75 | NA | NA |
| 2016 | maski | 106 | 169 | 282 | 113 | 1.75 | NA | NA |
| 2016 | maski | 107 | 161 | 273 | 112 | 5.75 | NA | NA |
| 2016 | maski | 120 | 155 | 274 | 119 | 4.25 | NA | NA |
| 2016 | maski | 121 | 146 | 261 | 115 | 3.5 | NA | NA |
| 2016 | maski | 122 | 156 | 269 | 113 | 3 | NA | NA |
| 2016 | maski | 123 | 148 | 275 | 127 | 4.25 | NA | NA |
| 2016 | maski | 124 | 157 | 261 | 104 | 5 | NA | NA |
| 2016 | maski | 125 | 152 | 276 | 124 | 5 | NA | NA |
| 2016 | maski | 126 | 158 | 281 | 123 | 2.25 | NA | NA |
| 2016 | maski | 127 | 151 | 244 | 86 | 2.5 | NA | NA |
| 2016 | maski | 200 | 166 | 290 | 124 | 2.25 | NA | NA |
| 2016 | maski | 201 | 170 | 320 | 150 | 1.75 | NA | NA |
| 2016 | maski | 202 | 167 | 282 | 115 | 3.25 | NA | NA |
| 2016 | maski | 203 | 166 | 279 | 113 | 7.25 | NA | NA |
| 2016 | maski | 204 | 181 | 276 | 95 | 3.5 | NA | NA |
| 2016 | maski | 205 | 172 | 273 | 101 | 5.25 | NA | NA |
| 2016 | maski | 207 | 172 | 326 | 154 | 3.25 | NA | NA |
| 2016 | maski | 208 | 168 | 260 | 92 | 6.25 | NA | NA |
| 2016 | maski | 209 | 174 | 263 | 89 | 6.5 | NA | NA |
| 2016 | maski | 210 | 170 | 274 | 104 | 8.75 | NA | NA |
| 2016 | maski | 211 | 162 | 274 | 112 | 6 | NA | NA |
| 2016 | maski | 212 | 165 | 272 | 107 | 5.25 | NA | NA |
| 2016 | maski | 213 | 170 | 272 | 102 | 5.5 | NA | NA |
| 2016 | maski | 214 | 170 | 271 | 101 | 5.75 | NA | NA |
| 2016 | maski | 215 | 161 | 272 | 111 | 4.25 | NA | NA |
| 2016 | maski | 216 | 173 | 291 | 118 | 2.5 | NA | NA |
| 2016 | maski | 217 | 161 | 268 | 107 | 3.75 | NA | NA |
| 2016 | maski | 218 | 165 | 256 | 91 | 3.25 | NA | NA |
| 2016 | maski | 219 | 161 | 263 | 102 | 3.5 | NA | NA |
| 2016 | maski | 220 | 157 | 269 | 112 | 6.5 | NA | NA |
| 2016 | maski | 221 | 169 | 297 | 128 | 1 | NA | NA |
| 2013 | tortue | 14 | NA | 261 | NA | 4.75 | NA | NA |
| 2013 | bog | 47 | NA | 252 | NA | 8.5 | NA | NA |
| 2013 | bog | 50 | NA | 269 | NA | 7.5 | NA | NA |
| 2014 | scirbi | 34 | NA | 260 | NA | 8.5 | NA | NA |
| 2014 | bog | 47 | NA | 262 | NA | 8 | NA | NA |
| 2014 | umiujaq | 61 | NA | 265 | NA | 1 | NA | NA |
| 2014 | umiujaq | 63 | NA | 258 | NA | 3.75 | NA | NA |
| 2015 | bog | 47 | NA | 259 | NA | 7.25 | 3.9675 | 45.075 |
| 2015 | umiujaq | 75 | NA | 255 | NA | 5.5 | 5.65 | 66.075 |
| 2015 | umiujaq | 76 | NA | 258 | NA | 3 | 5.29 | 85.25 |
| 2015 | umiujaq | 77 | NA | 261 | NA | 7.5 | 5.97 | 67.575 |
| 2015 | umiujaq | 78 | NA | 257 | NA | 6.5 | 5.8 | 67.25 |
| 2015 | umiujaq | 80 | NA | 261 | NA | 6.5 | 5.66 | 51.7 |
| 2015 | umiujaq | 84 | NA | 266 | NA | 3.5 | 5.5 | 73.8 |
| 2015 | maski | 120 | NA | 275 | NA | 4.66 | 6.41 | 45.375 |
| 2015 | maski | 121 | NA | 268 | NA | 3 | 6.405 | 37.225 |
| 2015 | maski | 123 | NA | 273 | NA | 3.66 | 6.62 | 56.225 |
| 2015 | maski | 124 | NA | 262 | NA | 4.33 | 6.52 | 57.575 |
| 2015 | maski | 125 | NA | 251 | NA | 4 | 6.225 | 52.2 |
| 2015 | maski | 126 | NA | 268 | NA | 2.33 | 6.09 | 42.375 |
| 2015 | maski | 201 | NA | 259 | NA | 1.33 | 5.865 | 85.5 |
| 2015 | maski | 202 | NA | 270 | NA | 2.66 | 5.895 | 86.65 |
| 2015 | maski | 203 | NA | 261 | NA | 8.66 | 6.13 | 61.225 |
| 2015 | maski | 205 | NA | 248 | NA | 6.33 | 6.185 | 52.95 |
| 2015 | maski | 207 | NA | 256 | NA | 2 | 6.09 | 61.525 |
| 2015 | maski | 208 | NA | 259 | NA | 7.33 | 6.045 | 62.5 |
| 2015 | maski | 209 | NA | 253 | NA | 4 | 6.235 | 57.675 |
| 2015 | maski | 210 | NA | 260 | NA | 11.33 | 6.22 | 59.225 |
| 2015 | maski | 211 | NA | 255 | NA | 6.33 | 6.36 | 61.5 |
| 2015 | maski | 212 | NA | 258 | NA | 5 | 6.61 | 61.925 |
| 2015 | maski | 213 | NA | 250 | NA | 3.66 | 6.46 | 37.7 |
| 2015 | maski | 214 | NA | 260 | NA | 6.33 | 6.665 | 56 |
| 2015 | maski | 215 | NA | 276 | NA | 5.66 | 6.32 | 16.9 |
| 2015 | maski | 216 | NA | 267 | NA | 1.66 | 6.515 | 24.625 |
| 2015 | maski | 219 | NA | 258 | NA | 3.33 | 7.005 | 42.725 |
| 2015 | maski | 220 | NA | 256 | NA | 6.66 | 6.815 | 54.75 |
| 2016 | scirbi | 23 | NA | 263 | NA | 9 | NA | NA |
| 2016 | tortue | 15 | NA | 274 | NA | 4.5 | NA | NA |
| 2015 | tortue | 13 | NA | NA | NA | 5.75 | 3.485 | 83.925 |
| 2015 | bog | 44 | NA | NA | NA | 6.75 | 3.9625 | 33.85 |
| 2015 | maski | 106 | NA | NA | NA | 1.33 | 6.62 | 50.475 |
| 2015 | maski | 107 | NA | NA | NA | 5.33 | 6.78 | 57.6 |
| 2015 | maski | 122 | NA | NA | NA | 4 | 6.395 | 62.775 |
| 2015 | maski | 127 | NA | NA | NA | 1.66 | 6.465 | 59.2 |
| 2015 | maski | 200 | NA | NA | NA | 7 | 5.985 | 89.05 |
| 2015 | maski | 204 | NA | NA | NA | 2.66 | 6.47 | 40.725 |
| 2015 | maski | 217 | NA | NA | NA | 3.33 | 6.555 | 45.525 |
| 2015 | maski | 218 | NA | NA | NA | 3 | 6.055 | 59.35 |
| 2015 | maski | 221 | NA | NA | NA | 1 | 6.025 | 51.375 |
| 2014 | tortue | 3 | NA | NA | NA | 1 | NA | NA |
| 2013 | tortue | 3 | NA | NA | NA | 1 | NA | NA |
| 2013 | bog | 53 | NA | NA | NA | 3.75 | NA | NA |
| 2013 | bog | 56 | NA | NA | NA | 2.75 | NA | NA |
| 2013 | umiujaq | 68 | NA | NA | NA | 5.5 | NA | NA |
| 2013 | umiujaq | 69 | NA | NA | NA | 4.75 | NA | NA |
